# Role of organizational commitment in career growth and turnover intention in public sector of Oman

**DOI:** 10.1371/journal.pone.0265535

**Published:** 2022-05-12

**Authors:** Adil Khamis Al Balushi, Venkat Ram Raj Thumiki, Nishad Nawaz, Ana Jurcic, Vijayakumar Gajenderan

**Affiliations:** 1 University of Technology and Applied Sciences (Higher College of Technology), Muscat, Oman; 2 Modern College of Business & Science, Muscat, Oman; 3 Department of Business Management, College of Business Administration, Kingdom University, Riffa, Bahrain; 4 School of Engineering Management, University ’Union—Nikola Tesla’, Belgrade, Serbia; 5 Sir Theagaraya College, Chennai, India; FAME|GRAPE, POLAND

## Abstract

**Introduction:**

Creating a proper career program is the best way to enhance employees’ organizational commitment; it motivates and retains them. This research aims to measure career growth’s influence on turnover intention, mediated by employees’ commitment through self-reported employees’ perceptions. This study identifies the key dimensions of organizational commitment (affective, continuance and normative commitment) that mediate the relationship between career growth and employee turnover intention exploring the indirect effects between career growth and turnover intention. The relationship is examined among the public sector employees in the Sultanate of Oman, a sector currently facing high turnover rates and losing key skilled talent pool.

**Methodology:**

Data collection was executed through an adopted questionnaire distributed among 500 employees of 38 government units within the Sultanate of Oman. A total of 351 questionnaires were returned, and after the initial screening process, 329 were found to be valid for further analysis. CFA analysis was conducted to identify the factors falling under the three primary constructs of the study. Assessment of the models was explained through Goodness-of-fit Indices. Structural equation modeling, which is most recommended to study the effect of latent variables, was performed using AMOS to evaluate the mediating role of organizational commitment between career growth and employee turnover intention.

**Findings:**

The results indicated that the potential for career growth is an essential motivating element for public sector employees in the Sultanate of Oman to encourage retention and reduce intention to leave. The findings also confirm that effective and continuous commitment significantly mediates the relationship between career growth and employees’ turnover intention.

**Contribution and originality value:**

The results generated could help both researchers and those involved in public sector policy-making to understand how employee turnover intention is influenced by career growth and organizational commitment in the public sector in the Sultanate of Oman.

## 1. Introduction

Any governmental and commercial organization will be challenged to retain its most valuable employees. Learning organizations improve the performance of their employees and reduce their intention to leave [1]. According to [2], this competition continues to intensify however, the public sector with less advancement in its traditional practices [3] is at a disadvantage. But, the improvement can be noticed in current managerial practices [4]. Employees seek an apparent career growth in the organization, a perceived value that enhances their organizational commitment [5, 6], which may further discourage them from leaving the organization [7]. Thus, creating a proper career growth-path is essential to retain existing employees [8] and attracts potential talents and contributes to the recruitment function’s effectiveness [9]. The development and implementation of a career program cannot happen overnight; it requires a long-term commitment and a holistic approach in managing the workforce [10, 11] and demands effort and commitment on the part of top management [12], line managers and human resource personnel [13–16]. Also, the best practice of the concept of career growth strategy may not be suitable to every environment and must be adopted to fit cultural contexts.

Omani economy functions on both public and private sectors. Private sector includes for profit organizations such as retail, commercial establishments, manufacturing sector, hotel industry, hospitality industry and private health care. Whereas the public sector of Oman inlcudes, Government institutions such as the Central Bank of Oman, Government Authorities such as Public Authority for Water, Government-owned companies such as Mwasalat, along with several offices serving health, agriculture, tourism, etc. [17]. Public sector in Oman is highly significant in terms of contribution to the economy and has a manpower of around 229,386 actively servng the sector by end of 2020 [18].

Employee retention has become a considerable challenge to the public sector in the Sultanate of Oman. It has been losing out on key technical and management personnel [19, 20], thus creating a severe talent gap in the sector.

## 2. Literature review

A systematic literature review was conducted to understand the problem and formulate research hypotheses. This section is divided into four parts: career growth, employee turnover intention, organizational commitment and relationships between the study variables.

### Career growth

Career growth is the progression of a career, related prospects, experiences, duties, and responsibilities through intervention and purposeful responsibility of individuals and their environment [21, 22]. Previously, many aspects of career growth have been studied, such as career self-management, employees’ early experiences in an organization [23, 24], and their urge for promotion [25, 26]. Though career development is sometimes used interchangeably with career growth [27], the former term is more concerned with the career plans structured and managed by employees and organizations [28, 29]. As the study of Tabachnick and Fidell has dubbed as an alternative to the career growth concept, the concept of career ladder explains that employees gain skills and upgrade their performance by efforts to improve their positions [30]. Career planning, an essential requirement in career growth, is how individuals and organizations identify their skills, opportunities, and interests as well as work towards achieving their career objectives [31]. The literature on career growth specifies that promote equity and job rotation are important sub-constructs in measuring career growth [32–36].

Career growth encompasses four essential components: career goal progression, professional ability development, promotion speed and remuneration growth. Employees feel an affinity towards their organization if it provides an environment that supports their career goal progression [37, 38]. Employees feel that their employers are committed to their career progression if they foresee a clear perspective about their future [39]. It positively impacts their organizational commitment [26]. Professional ability development refers to the deliberate actions by employers aimed at developing abilities, skills and knowledge required by employees to perform their day-to-day tasks [40–44]. Available literature indicates that professional ability development can impact organizational commitment [45–47]. As a piece of evidence for employers’ effective HRM strategies, such programs encourage reciprocity between two parties and ultimately result in creating employees’ emotional attachment and loyalty to their organization [48, 49]. Promotion speed refers to the time taken in ascending the company’s positional ladder [50]. It essentially involves an increase in job scope and responsibilities [51], which is usually treated as a visible organization-wide sign of achievement [52]; 2019; [53]. Remuneration growth is an essential component of career growth [26] that, according to the Social Exchange Theory, includes fair pay for the job done [54, 55] along with benefits, which ultimately attract the employees’ attention towards improved employee retention [56].

### Employee turnover intention

Turnover intention refers to an employee’s intention to leave his or her current organization [57], which is essentially a psychological process [58] wherein an employee considers leaving or resigning as well as searching for other work [57]. It can be identified in the withdrawal behavior of the employees. This would include work withdrawal, which refers to avoiding aspects of work, and job withdrawal, which refers to non-participation in working situations [59, 60]. Turnover intention shows a breach in the relationship between employees and their employers [61].

### Organizational commitment

Organizations need to build policies and practices to strengthen organizational commitment and raise employee job satisfaction [62]. Organizational commitment is a driving force for the employees to achieve mutual respect between them and their organization [63]. Employees exhibit organizational commitment when they perceive their organization’s commitment to individuals’ progression and achieve its objectives, which creates the expected effect [64]. According to [65, 66], empowering leadership will enhance employee organizational commitment. Premised upon the social exchange theory, organizational commitment can be divided into three types: affective commitment, continuance commitment and normative commitment [67]. Affective commitment is based on employers’ care and responsibility for their employees. Continuance commitment is based on losing the incentives and seniority if the employees were to leave because they are perceived as essential. Moreover, the final component, normative commitment, is when employees feel grateful and are obliged to be in their organization as a member.

### Relationship between career growth and employee turnover intention

Available literature recommends focusing on career growth components as a strategy to reduce turnover intention [68] as career growth is more closely related to employees’ behaviors and attitudes [26, 69] and can retain employees who are looking to develop their career [70]. In the previous studies, it is emphasized that low turnover can be affected by the internal career advancement opportunity perceived by the employees [71–73] and perceived fairness in terms of awards and promotion [74, 75]; Job rotation as a career growth strategy can cause job satisfaction [76] and promote retention [77–80]. A study used the model (which is adopted in this research) [26] and found that career growth correlated negatively with turnover intention. Along with [81], many studies used this model to examine turnover intention and organizational commitment. For example, a study done in Turkey by Karavardar [27] found that remuneration growth and professional ability development have a strong negative impact on turnover intention. Similarly, according to [53], there is a negative association between turnover and promotion speed.

### Relationship between career growth and organizational commitments

Researchers found that career growth components, such as professional development opportunity, promotion speed and remuneration growth, have been positively associated with employees’ commitment [44, 82–86], as the committed employees are more likely to participate in the organizational improvement [87, 88]. Organizations must offer opportunities for career advancement [89] as professional development strategies contribute to career growth which in turn can contribute to employee commitment [10, 46], particularly to continuance commitment. Promotion speed also may improve employees’ performance [90], as it is more likely to develop a sense of belonging and enhance job satisfaction as well [53, 91–93]. Another factor, remuneration growth or attractive compensation structure, can lead to a positive employee perception of their remuneration, which in turn increases organizational commitment [5, 81, 84].

### Relationship between employee turnover intention and organizational commitment

According to [86], organizations must devise strategies to enhance employee commitment to reduce turnover, because a negative correlation has been found between these two variables [94]. According to [5], organizational commitment is an important element that negatively affects turnover intention than other elements, such as career growth. Similar findings are also reported by [95], who studied call center workers. According to [96], the relationship between affective commitment and turnover intention is negatively related.

### Relationship between three variables

Proposed hypothesis of the model that was presented in Fig 2 attempts to explain the relationship between the study variables. The hypothesis model was proposed based on the literature review. Available literature [26, 44, 73, 81, 97] indicates that organizational commitment is positively impacted by the potential for career growth and negatively impacted by turnover intention. Further, a detailed analysis of setting up of hypotheses was expalined on page numbers ……… clearly explaining the relationship between career growth and affective commitment (H1), continuance commitment (H2), normative commitment (H3) along with turnover intension (H4). Hypotheses explaining the relationship between organizational commitment and turnover intention are set from H5 to H7. Mediating role of organizational commitment was explained through hypotheses, H 8 to H10.

### Need for the study

In spite of several studies conducted to test career growth and organizational commitment connecting with the turnover intention [15, 26, 81], insufficient attention has been paid to understand the public sector [44]. The ample empirical research on career growth, organizational commitment and turnover intention that has been conducted in Western countries [98, 99] and Eastern countries [100–106] may not have much validity in the Middle Eastern context [107]. As such, more research is required to understand how people in different parts of the world conceptualize and understand individual behaviors that encourage them to continue their work within their current organizations. According to [81], most literature reviews in this area do not include all organizational commitment components. Hence, to fill the research gap, this study has tested all three components of organizational commitment and how they mediated the relationship between career growth and turnover intention, thus contributing to the theoretical robustness of the model originally proposed by [26].

According to [20], who conducted a qualitative study in Oman, organizational commitment is a major theme that indicated diminished normative commitment. This reinforces the need for further exploratory testing of all three components of organizational commitment quantitatively. Finally, in terms of practical significance, there are currently a limited number of studies that have been conducted on this aspect specifically focused on Oman’s public sector [108–110]. According to [111], because of economic growth in the GCC region, there is strong competition among public-sectors of the member countries regarding employees’ skills, which are negatively affected by turnover. This study attains significance in this scenario by addressing turnover intention in the public sector of Oman to discover the reasons behind individual attrition.

### Objective of the study

This research aims to examine the mediating role of organizational commitment that influences the impact of career growth on the turnover intention in the public sector of the Sultanate of Oman.

### Research methodology

This descriptive research was conducted with the help of the survey method [112, 113], as this approach seemed to be an optimal alternative to other data collection methods because it poses straightforward and standard questions at a homogeneous sample [114] and enables data analysis [115].

### Description of population and sampling

The target population for this study comprises the permanent employees working in the public sector in the Sultanate of Oman with at least three years of service. The reason behind choosing three years’ experience is to ensure that the employees are entrenched in their career and not new employees. Employees located in selected Ministries in the Government were chosen as respondents to accomplish the study’s aims. The survey targeted active employees in 38 Ministries in the Sultanate of Oman with at least three years of service. A stratified convenience sampling method was used to select the respondents for this study. A total of 500 survey questionnaires were distributed to public sector employees—out of which 351 questionnaires were returned. Furthermore, after initial screening, 329 valid questionnaires were considered for the study.

### Determining the adequacy of sample size

According to [116], various aspects, such as cost, time and resource availability, should be considered while determining the sample size. Furthermore, it is important to consider the effects that the sample size has on the statistical accuracy of the research [115]. As there is no real science when selecting the sample size [117], the sample size for this research was based on what past researchers have recommended. A suitable sample size regarding to [30] is based on the ratio of 1:40, which means for each variable of the study, there have to be at least 40 samples if the study employs a regression analysis. Based on this rule, the recommended sample size should be a minimum of 320 (8 x 40) cases. Additionally, another argument by [118] discussed that six independent variables could be calculated as 104 + 6 = 110 cases that may need to exam individual predictors, plus 50 + (8) (6) = 98 cases to exam the regression. However, [115] noted that every research is unique, and thus, has different requirements. Therefore, the sample size should be based on the research requirements rather than on the statistical assumptions [115]. Furthermore, according to [119], the basic rule of thumb on determining the adequate sample size. Based on the various assumptions and guidelines provided by the previous researchers [115, 120], this study has chosen the large number that can rely and ensure the minimum cases needed for successful structural equation modelling (SEM).

### Questionnaire design

For a questionnaire survey to be done successfully, the questions need to be straightforward, the layout should be clear, and the sequence should be easy to follow [120]. Additionally, the questionnaire should be able to translate the information that is needed and be entertaining so as not to cause the respondent to be bored. Finally, it should seek to reduce response error [117]. The questionnaire for this research was designed based on an extensive literature review. An adapted version of the questionnaire was used in this study. However, a few items were also modified accordingly to fulfill the purpose of the research interest and to fit the Omani context. Though the questionnaire was developed in English, keeping in mind concerning language barrier, it is translated into Arabic. A five-point Likert scale was used to scale rangingfrom ‘strongly disagree’ (1) to ‘strongly agree’ (5). Section one consisted of demographic information, and section two consisted of problem-related variables identified for this study.

### Variables considered for the study

Along with the demographic variables, 31 problem-related variables were considered for study based on the literature (Table 1). They are further divided into three constructs: career growth, organizational commitment and employee turnover based on the literature (Table 2). Four sub-constructs that describe career growth are career goal progress (variables 1 to 4), professional ability development (variables 5–8), promotion speed (variables 9–12) and remuneration growth (variables 13–15). Three sub-constructs that describe organizational commitment are affective commitment (variables 16–19), continuance commitment (variables 20–23) and normative commitment (variables 24–28). Finally, employee turnover intension is described by variables 29–31.

**Table 1:** Variables considered for the study.

No	Variable
1	My present job moves me closer to my career goals.
2	My present job is relevant to my career goals and vocational growth.
3	My present job sets the foundation for the realization of my career goals.
4	My present job provides me with good opportunities to realize my career goals.
5	My present job encourages me to continuously gain new and job-related skills.
6	My present job encourages me to continuously gain new job-related knowledge.
7	My present job encourages me to accumulate richer work experiences.
8	My present job enables me to continuously improve my professional capabilities.
9	My promotion speed in the present organization is fast.
10	The probability of being promoted in my present organization is high.
11	Compared with previous organizations, my position in my present one is ideal.
12	Compared with my colleagues, I am being promoted.
13	My salary is growing quickly in my present organization.
14	In this organization, the possibility of my current salary being increased is large.
15	Compared with my colleagues, my salary has grown more quickly.
16	I would be very happy to spend the rest of my career with this organization.
17	I do not feel a strong sense of belonging to my organization.
18	This organization has a great deal of personal meaning for me.
19	I really feel as if this organization’s problems are my problems.
20	Right now, staying with my organization is a matter of necessity as much as the desire.
21	It would be very hard for me to leave my organization right now, even if I wanted to.
22	Too much of my life will be disrupted if I want to leave my organization now.
23	I feel that I have too few options to consider leaving this organization.
24	Even if it were to my advantage, I do not feel it would be right to leave my organization now.
25	I would feel guilty if I left my organization now.
26	This organization deserves my loyalty.
27	I would not leave my organization right now because I have a sense of obligation to the people in it.
28	I do not feel any obligation to remain with my current employer.
29	I am very likely to stay in this company for the next five years.
30	I will not give up this company easily.
31	For me, this company is the best of all possible organizations to work for.

**Table 2:** Distribution of variables into constructs based on the literature.

Construct	Sub-construct	Variables	Reliability	Literature
Career growth	Career goal progress	1–4	0.85	[8]
	Professional Development Ability	5–8	0.86	
	Promotion speed	9–12	0.8.	
	Remuneration growth	13–15	0.78	
Organizational commitment	Affective commitment	16–19	0.86	[102]
	Continuance Commitment	20–23	0.84	
	Normative Commitment	24–28	0.78	
Employee Turnover Intention	Employee Turnover Intention	29–31	0.70	[121]

### Data analysis tools

This study employed various analyses, including demographic, descriptive, reliability as well as validity testing. Using AMOS software Version 21, descriptive statistics were used to identify the importance of individual variables, Confirmatory Factor Analysis was used to group variables into factors and constructs, and Structural Equation Modelling was employed for testing the hypotheses to prove the mediating role of organizational commitment between career growth and turnover intention. Hypotheses were formulated after a careful review of literature [117].

### Setting of hypotheses

#### Career growth and affective commitment

Affective Commitment occurs when an organization fulfills primary needs of its employees [62], and thus the employee identifies himself or herself with the organization’s goals and strives to facilitate the fulfillment of the goals [122]. Hence, as a reciprocal response, the organizations must meet the employees’ career goals and contribute to their career growth [123]. Thus, to propose the first hypothesis in this research, we say that the experience of the employees in career growth must have a higher level of affective commitment. Firstly, employees must be working in a field that is related to their career goals. Secondly, it must make room for professional growth, which allows them to gain new skills. Thirdly, the reward and promotion speed scheme that employees perceive has to be responsive to their efforts.

**Hypothesis 1**: There is a significant relationship between career growth and affective commitment in the Omani public sector.

#### Career growth and continuance commitment

Though the disadvantage of discontinuing work and the cost of leaving may contribute to continuance commitment [124], according to [102], employees’ opinion to remain in their organization is based mainly on their sense of commitment to their employers. Other reasons include personal level status, remuneration, seniority and other benefits that employees acquired from their organization that contributed to their career growth. Thus, career growth can be seen as a great predictor of continuance commitment. According to [41], gaining new skills at the current job has the potential to create continuance commitment. When organizations provide opportunities for skill acquisition, it builds exit barriers. Thus, employees consider continuing in their present organization. Thus, the following hypothesis is proposed for this study:

**Hypothesis 2**: There is a significant relationship between career growth and continuance commitment in the Omani public sector.

#### Career growth and normative commitment

According to [102], normative commitment refers to the employee’s psychological attachment to the organization based on either socialization experiences that emphasize the appropriateness of remaining loyal or a moral obligation to repay the organization for benefits received. According to [105], normative commitment indirectly influences an affective commitment to some relational variables, such as employee satisfaction, solidarity and participation in decision making and employee trust in the firm. Hence, the normative commitment as a mind-set of obligation is worthy of continuing investigation, as this is different from the mindset of desire [103], being faithful to the organization [98] and the individual’s sense of obligation [125]. Therefore, from the discussion above, researchers propose the following hypothesis:

**Hypothesis 3**: There is a significant relationship between career growth and normative commitment in the Omani public sector.

#### Career growth and turnover intention

According to [126], when the employee seeks an opportunity for career growth, he or she has a high expectation from the employer. Therefore, the degree of remaining within the same organization depends on how the employer has met their desire of career growth. Conversely, if the organization fails to meet its employees’ career growth expectations, it drives employees to seek opportunities elsewhere, which may be more suitable for their employment expectations. The literature indicates a positive relationship between career growth and organizational commitment and a negative relationship with the turnover intention [26, 126]. On the contrary, failure to provide career growth opportunities would result in employees—particularly the talented ones-searching for opportunities elsewhere that is more attractive. According to [53], there is a negative association between turnover and promotion plus salary growth. This research aims to examine closely and look at the high predictors of career growth factors’ effect on turnover intention that may help to explain further the connection among them. Thus, the following hypothesis was set:

**Hypothesis 4**: Career growth is negatively associated with turnover intention in the Omani public sector.

#### Organizational commitment and turnover intention

There are numerous studies [127–129], which reported that organizational commitment is connected closely to turnover intention. The employees’ intention for leaving the organization is considered based on their personal attachment to the organization; it is less likely for those who have a higher attachment to their organization. This research will focus on exploring the strongest link with turnover intention. So, the following hypotheses would propose:

**Hypothesis 5**: There is a significant negative relationship between affective commitment and turnover intention in Omani’s public sector.
**Hypothesis 6**: There is a significant negative relationship between continuance commitment and turnover intention in Omani’s public sector.
**Hypothesis 7:** There is a significant negative relationship between normative commitment and turnover intention in Omani’s public sector.

#### Mediating role of organizational commitment

According to [126], the reason behind individuals’ high sense of duty regarding their vocation/occupation will expect more from their organization and how much those desires are met will determine their work relationship. Studies carried out by [95, 130–132] indicated that organizational commitment affects turnover intention more negatively than career growth. According to [96], the relationship between the component of affective commitment and turnover intention is a negatively related. In this case, this research will examine whole commitment components, including affective occupational commitment and turnover intentions. This study aims to examine the relationship between career growth and turnover intention as well as to examine the mediating role of organizational commitment. So, the following hypothesis would propose:

**Hypothesis 8**: Affective commitment mediates the relationship between career growth and turnover intentions.
**Hypothesis 9**: Continuance commitment mediates the relationship between career growth and turnover intentions.
**Hypothesis 10**: Normative commitment mediates the relationship between career growth and turnover intentions.

### Proposed conceptual framework

**Fig 1:**
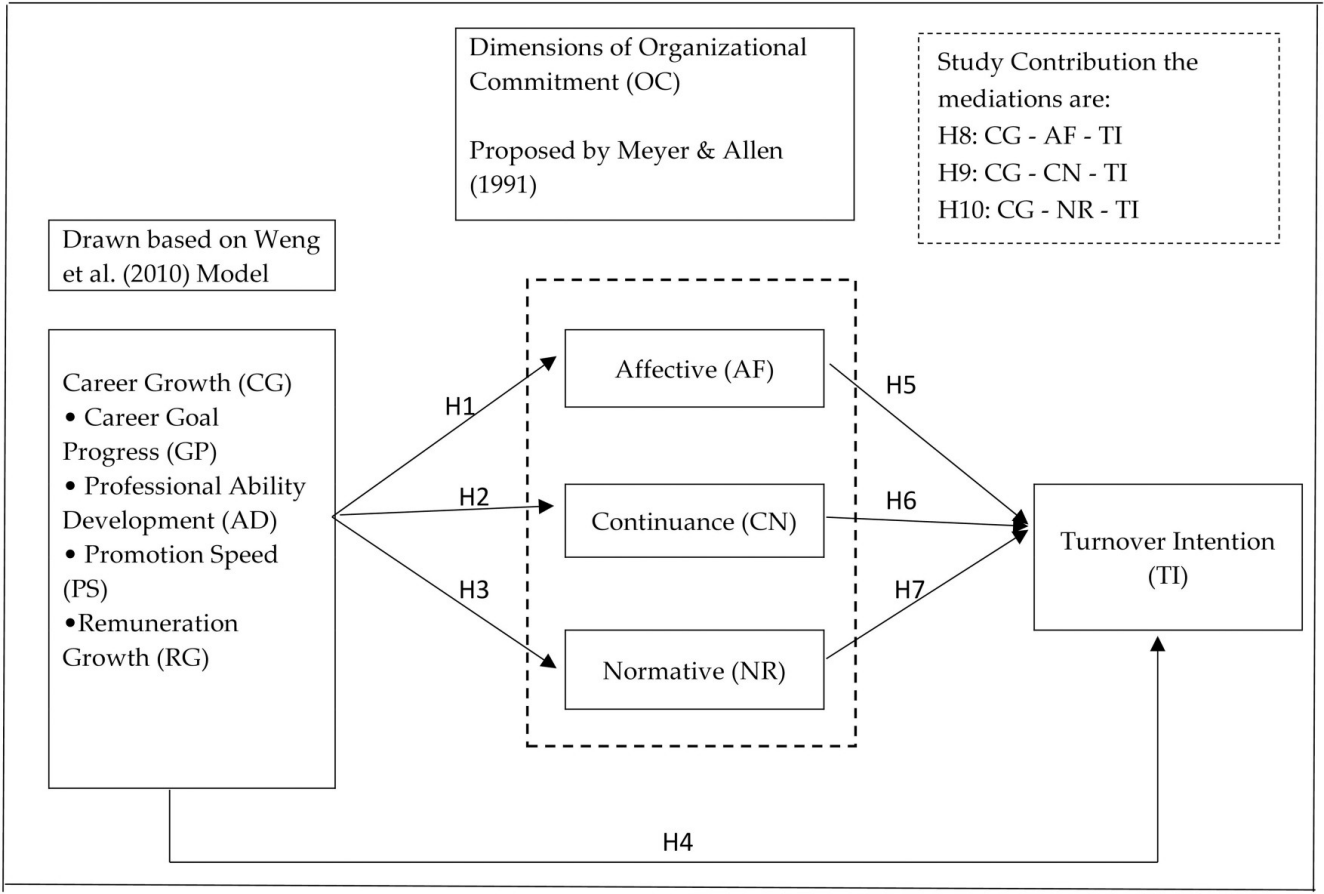
Proposed conceptual framework. Source: Authors’ adaptation.

Based on the above discussion, the following conceptual framework is proposed (Fig 1). The research has both dependent and independent variables and mediator variables, as shown in the figure below.

### Reliability and validity analysis for Confirmatory Factor Analysis

#### Validity

For discriminant validity, CFA was conducted for career goal progresses, professional ability development, promotion speed, remuneration growth, affective commitment, continuance commitment, normative commitment and employee turnover intention. To achieve the discriminant validity, this study runs the measurement model by linking all the exogenous and endogenous constructs to examine whether these constructs are highly correlated. In the case where the measure of correlation between two constructs is higher than 0.85, one could conclude that the discriminant validity is not achieved [133, 134]. If the discriminant validity is not achieved, then the researchers need to drop one of those two constructs for further analysis since it mirrors the other [133–135]. Table 3 indicates that none of the exogenous constructs’ correlation is higher than 0.85. Thus, it is assumed that the discriminant validity is achieved.

**Table 3:** Correlations: (Default model).

		Estimate
Carrier Growth	Affective Commitment	0.336
Carrier Growth	Continuance Commitment	0.504
Carrier Growth	Normative Commitment	0.488
Carrier Growth	Employee Turnover Intention	-0.328
Affective Commitment	Continuance Commitment	0.187
Affective Commitment	Normative Commitment	0.177
Affective Commitment	Employee Turnover Intention	-0.480
Continuance Commitment	Normative Commitment	0.278
Continuance Commitment	Employee Turnover Intention	-0.297
Normative Commitment	Employee Turnover Intention	-0.239

#### Reliability

Reliability analysis using Cronbach’s Alpha needs to be conducted in factor analysis, as it enables measuring the internal consistency of variables grouped in each factor [136]. The value of Cronbach’s Alpha should be as close as possible to 1.0 or reasonably, between 0.7 and 1.0. A higher number indicates a higher correlation among the variables in the model. As presented in Table 4, the Cronbach’s Alpha value for all 31 items is 0.822 and greater than 0.7 for each of the factors or constructs. This indicates the significance of the model used in the analysis [137]. Table 5 summarizes the fulfillment of unidimensionality, reliability and validity requirements for the CFA analysis. The CFA results passed the unidimensionality, validity as well as reliability tests and allowed for further analysis.

**Table 4:** Reliability statistics of factors.

Variable	Cronbach’s Alpha	No of Items
Career Goal Progresses	0.805	4
Professional Ability Development	0.855	4
Promotion Speed	0.741	4
Remuneration Growth	0.766	3
Affective Commitment	0.929	4
Continuance Commitment	0.802	4
Normative Commitment	0.818	5
Employee Turnover Intention	0.933	3
Overall	0.822	31

**Table 5:** Reliability and validity of CFA measurement models.

Validity & Reliability	Name of category	Required value	Comments
**Validity**	Unidimensionality	Factor loading for each item ≥ 0.50	The required level is achieved (Table 8)
	Convergent Validity	Average Variance Extracted (AVE) ≥ 0.50	The required level is achieved (Table 8)
	Construct Validity	All fitness indices for the models meet the required level	The required level is achieved (Table 6)
	Discriminant Validity	Correlation between exogenous constructs is ≤ 0.85	The required level is achieved (Table 3)
**Reliability**	Internal Reliability	Cronbach’s alpha ≥ 0.70	The required level is achieved (Table 4)
	Construct Reliability (CR)	CR ≥ 0.60	The required level is achieved (Table 8)
	Average Variance Extracted (AVE)	AVE ≥ 0.50	The required level is achieved (Table 8)

It is important to note that Average Variance Extracted (AVE) and Composite Reliability (CR) are vital components that measure the convergent validity and composite reliability of the CFA model [133, 134]. However, one of the limitations of AMOS software is that it cannot calculate AVE and CR. Therefore, in this study, the AVE and CR were calculated based on the formula provided by [138].

AVE and CR Formula

$$CR=\frac{{\left(\sum \lambda \right)}^{2}}{\left[{\left(\sum \lambda \right)}^{2}+\sum (1-\lambda^{2})\right]}$$

$$AVE=\frac{\sum \lambda^{2}}{\left[\sum \lambda^{2}+\sum (1-\lambda^{2})\right]}$$

Source: [138]

### Normality and outlier analysis

AMOS software (version 21) was used to test the normality through the indices of skewness and kurtosis. The skewness for the measurement items should be either positive or negative to show that the assumption of normality is not violated [30, 139]. In this study, none of the constructs violated the assumption of normality as all the values of skewness were less than three, which were positively or negatively skewed [30]. Besides, for kurtosis, all the values should be less than seven to prove that there is no abnormal distribution [135]. All the kurtosis values in this study are less than five, proving that no single case falls in the abnormal distribution. Furthermore, from the output, the outliers were checked, and no observation was found on the Mahalanobis is distance (observations farthest from the centroid), presented in S1 Appendix. According to [133, 140], it is obvious that the values in the p2 column are always smaller compared to column p1. If the value in p1 column is smaller than p2 column, then the issue of outlier will exist. In this study, none of the values in p1 column were smaller than p2 column. This justifies the use of CFA analysis in this research.

### Structural equation modelling

Over the past few years, SEM has become a prevalent statistical techniques in research. In the past, SEM was mostly used by the specialist. However, due to the introduction of new SEM software that proves the user’s graphical user-interface, it is easy to use. The main advantage of SEM is flexibility; it proves the researcher with the ability to test a theoretical and measurement assumption against empirical data statistically. With the use of SEM, social scientists can now perform path analytical modelling with the use of latent variables [120]. It is important to highlight that SEM allows for the integration of unobserved (known as latent) variables with the observed ones and identifies structural causal links that arise between them [120]). Moreover, [141] stated that SEM is a confirmatory method, which means that the approach focuses on the hypothesis. While employing SEM, the theory is said to be a representation of the causal process that exists among multiple variables.

### Reliability analysis (Goodness-of-fit indices)

When using SEM, the researchers can test the model in a way that allows for the assessment of the models’ goodness-of-fit as well as whether the relationships between the variables are deemed acceptable or not [134]. However, there are multiple goodness-of-fit Indices that are used to validate the modelling (Table 6). Thus, the following goodness-of-fit Indices criteria was followed as suggested by the researchers [120, 134, 141–143].

**Table 6:** Goodness-of-fit indices.

Category	Index	Acceptance Level	Comments
Absolute Fit	Chisq	P>0.05	Sensitive to sample size >200
	RMSEA**	<0.08	Range 0.05 to 0.10 is acceptable
	GFI**	>0.90	>0.95 indicates a good fit
Incremental Fit	AGFI	>0.90	>0.95 indicates a good fit
	CFI**	>0.90	>0.95 indicates a good fit
	TLI	>0.90	>0.95 indicates a good fit
	NFI	>0.90	>0.95 indicates a good fit
Parsimonious fit	Chisq/df**	<3–5	<3 indicates a good fit

Source: [120, 134, 141]

**These indexes are recommended since these are highly reported in the literature.

### Ethics statement

The current research has adhered to the acceptable ethical standards in terms of institutional approval and data collection. Permission was obtained from the Institute of Public Administration, Oman for collecting data from public enterprise employees in Oman and to conduct the research. The questionnaire contained the Informed Consent Form that the respondents read before providing their responses to the survey. The information was gathered after ensuring confidentiality and legal implications. Both attachments (Approval letter & Informed Consent Form) are submitted separately to the journal. The study did not include minors. It is not a medical related survey.

## 3. Results and discussion

### Demographic analysis of sample data

Table 7 presents the demographic analysis of data collected from 329 respondents. The sample comprises around 63% are males and 37% females, and it matches the population characteristics. According to the National Centre for Statistics and Information (NCSI), 26.6% of the total workforce in public sector comprise of women [144]. The majority of the respondents (91.8%) are within 28 to 47 years. The data shows that majority of respondents are married, while only 18.5 percent of the respondents are unmarried (single). Finally, 54.7 percent of the respondents have a bachelor degree, while 41.9 percent have postgraduate degree. However, only 3.3 percent of the respondents have a certificate or diploma.

**Table 7:** Demographic analysis of sample data.

N = 329			
Demographic variable		No.	Percentage
Gender	Male	207	62.9
	Female	122	37.1
	Total	329	100
Age group	18–27 years	23	7
	28–37 years	132	40.1
	38–47 years	170	51.7
	48 years and above	4	1.2
	Total	329	100
Marital status	Single	61	18.5
	Married	268	81.5
	Total	329	100
Educational background	Certificate/Diploma	11	3.3
	Bachelor Degree	180	54.7
	Postgraduate	138	41.9
	Total	329	100

### Confirmatory Factor Analysis (CFA)

CFA results of this study include eight factors, career goal progress, professional ability development, promotion speed, remuneration growth, affective commitment, continuance commitment, normative commitment and employee turnover intention, categorized under four constructs, career growth, organizational commitment and turnover intention. The following section highlights the results obtained from the CFA analysis (Table 8) with the help of Factor loadings, Average Variance Extracted (AVE) and Composite Reliability (CR).

**Table 8:** CFA results.

Construct		Item	Loadings	AVE	CR
Career growth	Goal Progresses	My present job moves me closer to my career goals.	0.788	0.568	0.840
		My present job provides me with good opportunities to realize my career goals.	0.770		
		My present job sets the foundation for the realization of my career goals.	0.774		
		My present provides me good opportunities to realize my career goals.	0.678		
	Professional Development	My present job encourages me to continuously gain new job-related skills.	0.782	0.628	0.871
		My present job encourages me to continuously gain new job-related knowledge.	0.821		
		My present job encourages me to accumulate richer work experience.	0.787		
		My present job enables me to continuously improve my professional capabilities.	0.780		
	Promotion speed	My promotion speed in present organization is fast.	0.619	0.507	0.803
		The probability of being promoted in my present organization is high.	0.768		
		Compared with the previous organizations my position is my present one is ideal.	0.681		
		Compared with my colleagues, I am being promoted.	0.770		
	Remuneration	My salary is growing quickly in present organization.	0.793	0.601	0.819
		In this organization, the possibility of current salary being increased is large.	0.754		
		Compared with my colleagues, my salary has grown more quickly.	0.778		
Organizational commitment	Affective	I would be very happy to spend the rest of my career with this organization.	0.895	0.764	0.928
		I do not feel strong sense of belonging to my organization.	0.884		
		This organization has a great deal of personal meaning for me.	0.862		
		I really feel as if the organization’s problems are my problems.	0.855		
	Continuance	Right now, staying with my organization is a matter of necessity as much as desire.	0.777	0.587	0.851
		It would be very hard for me to leave my organization right now, even if I wanted to.	0.765		
		Too much of my life would be disturbed if, I want to leave my organization now.	0.755		
		I feel that I have too few options to consider leaving this organization.	0.768		
	Normative	I would feel guilty if I left my organization now.	0.629	0.520	0.843
		This organization deserves my loyalty.	0.813		
		I would not leave my organization right now because I have a sense of obligation to the people in it.	0.731		
		I do not feel any obligation to remain with my current employer.	0.743		
Turnover Intentions		You are very likely to say in this company for the next five years.	0.890		
		You will not give up this company easily.	0.865		
		For you, this company is the best of all possible organizations to work for.	0.886		

### Construct 1: Career growth

The first construct identified in this study is career growth. Four factors or sub-constructs were extracted under the career growth construct, which are career goal progresses, professional ability development, and promotion speed and remuneration growth. This study has confirmed that career growth is one of the most important issues influencing employee turnover intention. Fifteen items were adapted to measure the four sub-constructs of career growth. Career goal progress, professional ability development and promotion speed are measured by four items each, and remuneration growth is measured by three items. The item “My present job encourages me to continuously gain new job-related knowledge” has achieved the highest mean average of 4.8754 (Table 10). This means that employees working in the public sector of the Sultanate of Oman are encouraged to go for training regularly to ensure that their knowledge is current and related to their job.

On the other hand, the item “My promotion speed in the present organization is fast” has achieved the lowest mean average of 1.5957 (Table 10). This shows that most of the employees working in the public sector of the Sultanate of Oman are not happy with the promotion speed. This statistical information leads us to believe that these sub-constructs are among the main components, which determine career growth. This finding is consistent with past findings [27, 59], where the authors have identified these dimensions to measure career growth. Thus, it is vital to understand these sub-constructs of career growth, which may help the Omani government as well as the people involved in the ministry to develop better policies; this certainly would lead to retaining their loyal employees.

### Assessing the measurement model for career growth

**Fig 2:**
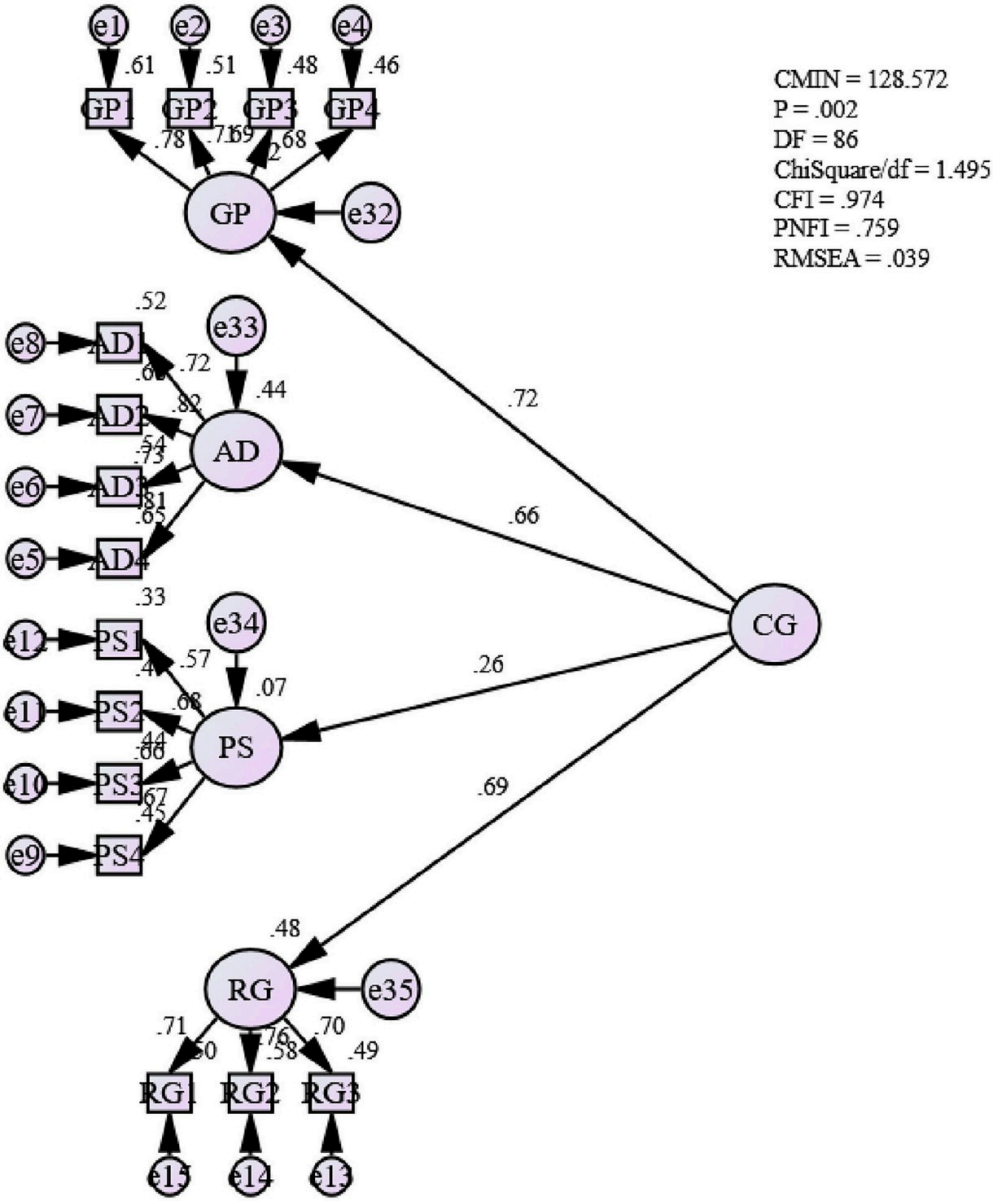
Measurement model for career growth.

Fig 2 shows the Goodness-of-fit indices (GOF) for the measurement model of career growth. It can be seen that the fitness level is achieved Absolute fit (RMSEA) = .039, (ChiSq/df) = 1.495; Incremental fit (CFI) = .974; and Parsimonious fit (PNFI) = .759]. Hence, this study assumes that the unidimensionality for the measurement model for ‘career growth’ has been achieved. Therefore, no more modification was needed for this model.

Table 9 also shows that all the path estimates for the higher order model is significant as p-value is ≤ 0.05 as suggested by Hair et al. (2016). This also confirms that all the career growth sub constructs (career goal progresses, professional ability development, promotion speed and remuneration growth) represents the actual constructs. Though the factor loading between career growth (CG) and promotion speed (PS) is low, 0.264, it is statistically significant to be included in the model. This justifies the use of higher order model (also known as second order model) for the construct career growth in this study.

**Table 9:** Regression weights.

		Estimate	SE	CR	p
Career goal progress	Career Growth	0.724	0.180	4.022	***
Professional ability development	Career Growth	0.662	0.156	4.234	***
Promotion speed	Career Growth	0.264	0.119	2.218	***
Remuneration growth	Career Growth	0.691	0.163	4.239	***

### Construct 2: Organizational commitment

Three factors were extracted under the organizational commitment construct, they are affective, continuance and normative commitment. Thirteen items were adapted to measure the sub-constructs of organizational commitment. Affective commitment and continuance commitment were measured by four items each, the normative commitment was measured by five items. The item “I do not feel any obligation to remain with my current employer” has achieved the highest mean average of 3.9289 (Table 10). This means that employees working in the public sector of the Sultanate of Oman are not satisfied with their current jobs. Therefore, they do not feel any obligation to remain in the organization. This was also supported by [20], who have, in an in-depth qualitative study, suggested diminished normative commitment. This is possibly due to the defaulted values of Islamic obligation and loyalty being challenged where autocracy thwarts trust from subordinates to superiors.

**Table 10:** Mean and standard deviation scores for each surveyed items.

	Mean and standard deviation scores for each surveyed items	Mean	SD
Career growth	My present job moves closer to my career goals	3.4863	0.90424
	My present job provides me with good opportunities to realize my carrier goals	3.4863	0.94706
	My present job sets the foundation for the realization of my carrier goals	3.6444	0.99907
	My present job provides me with good opportunities to realize my carrier goals	2.2705	0.7709
	My present job encourages me to continuously gain new and job related skills	3.769	0.79335
	My present job encourages me to continuously gain new and job related knowledge	4.8754	0.83354
	My present job encourages me to accumulate richer work experiences	3.997	0.81711
	My present job enables me to continuously improve my professional capabilities	3.9666	0.81269
	My promotion speed in the present organization is fast	1.5957	1.01698
	The probability of being promoted in my present organization is high	3.5684	0.93168
	Compared with my previous organization, my position in my present one is ideal	2.4863	0.95347
	Compared with my colleagues, I am being promoted	2.6292	1.00723
Organizational commitments	My salary is growing quickly in my present organization	2.2614	0.89973
	In this organization, the possibility of my current salary being increased is large	2.0365	0.94927
	Compared with my colleagues. My salary has been grown more quickly	2.2006	0.99505
	I would be very happy to spend the rest of my career in this organization	2.307	0.94674
	I do not feel a strong sense of belonging to my organization	1.1884	0.97892
	This organization has a great deal of personal meaning for me	3.2432	0.93793
	I really fell as if this organization’s problems are my problems	3.1307	0.98986
	Right now, staying with my organization is a matter of necessity as much as desire	3.2888	0.88615
	It would be very hard for me to leave my organization right now, even if I wanted to	2.2188	0.95036
	Too much of my life would be disrupted if I want to leave my organization now	2.2948	0.96018
	I feel that I have too few options to consider leaving this organization	1.3313	0.90542
	Even if it were to my advantage, I donot feel it would be right to leave my organization now.	2.6109	0.99419
	I would feel guilty I left my organization now	2.8237	1.01779
	This organization deserves my loyalty	2.614	1.00301
	I would not leave my organization right now because I have a sense of obligation to the people in it.	2.5897	1.00548
	I do not feel any obligation to remain with my current employer	3.9289	0.95951
	I am very likely to stay in this company for the next five years	3.245	0.98942
Turnover intention	I will not give up this company easily	3.7812	1.0509
	For me, this company is the best of all possible organization to work for	3.8906	0.99704

On the other hand, the item “I do not feel a strong sense of belonging to my organization” has achieved the lowest average mean score of 1.1884 (Table 10). This shows that majority of the employees working in the public sector of the Sultanate of Oman do not agree with this statement, as they prefer to work in their current organization. This is because they feel their current organization as their home and are currently happy with their career growth and future opportunities. This statistical information leads us to believe that these sub-constructs are among the main components determining organizational commitment. This finding is consistent with past findings [100, 101, 122] where the authors have identified these dimensions to measure organizational commitment. Thus, it is vital to understand these sub-constructs of organizational commitment, which may help the Omani government as well as the people involved in the ministry in implementing new policies and developing proper regulations that will create awareness among the various public sector authorities.

### Assessing the measurement model for affective commitment

Initially, it was found that the MI (Modification Index) value between e16 (AC1) and e19 (AC4) was more than fifteen. Thus, a double-headed arrow was used to demonstrate them to be “free parameter estimate”, and the model was re-specified.

**Fig 3:**
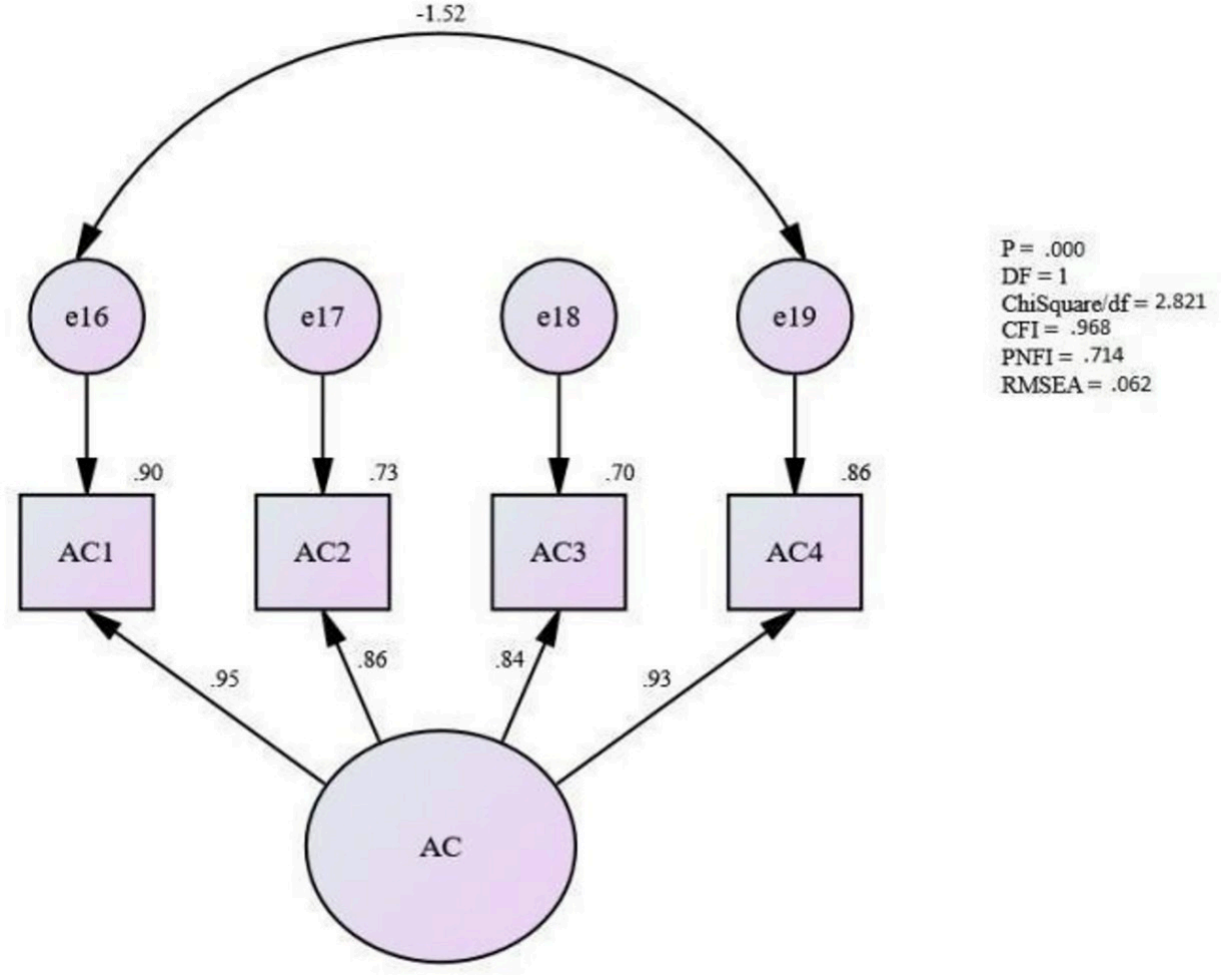
Measurement model for affective commitment.

After re-specifying the model and assessing the fitness level for the measurement model for quality (Fig 3), it shows that the fitness level for the measurement model for quality is achieved [Absolute fit (RMSEA) = .062, (ChiSq/df) = 2.821; Incremental fit (CFI) = .968; and Parsimonious fit (PNFI) = .714]. Hence, this study assumes that the unidimensionality for the measurement model for affective commitment has been achieved. Therefore, no more modification was needed for this model.

### Assessing the measurement model for continuance commitment

**Fig 4:**
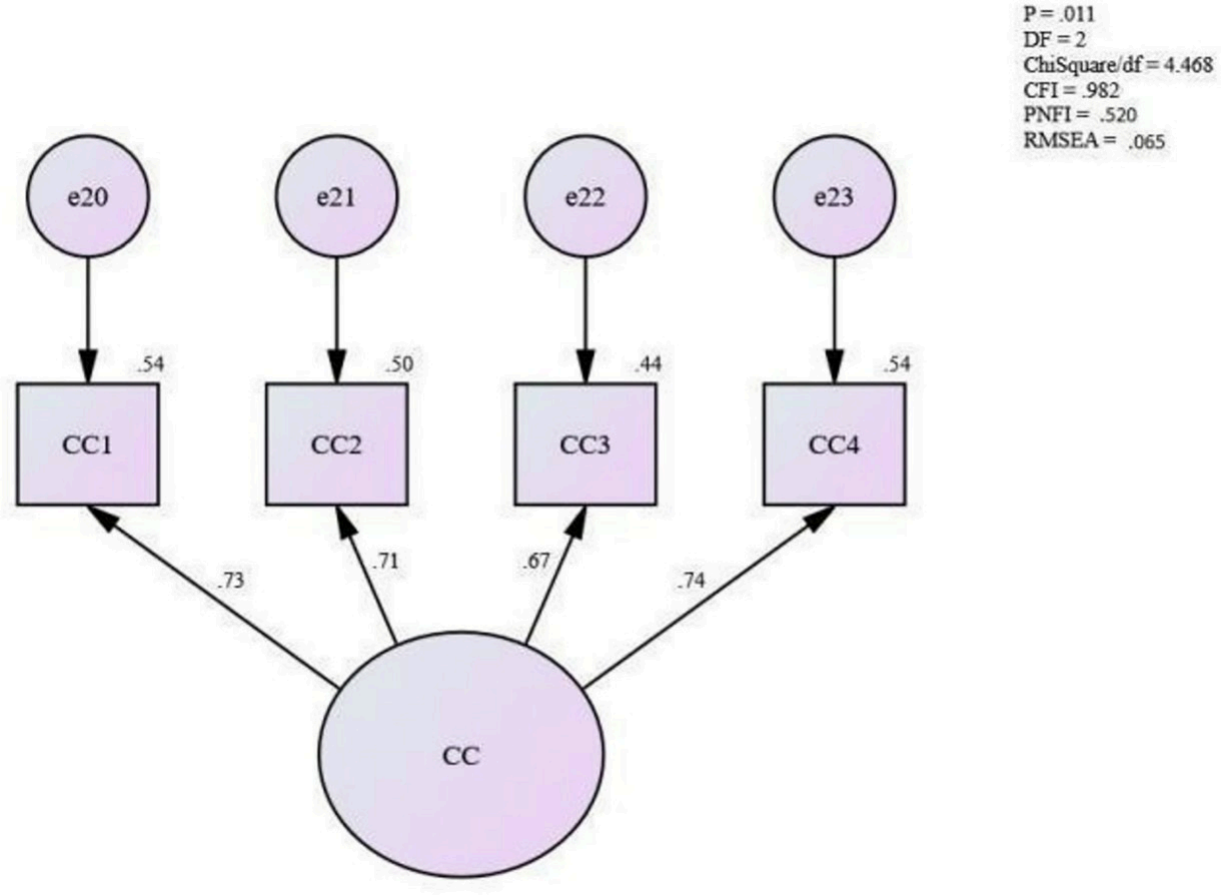
Measurement model for accessibility & availability of various types of treatments.

Fig 4 shows the measurement model for continuance commitment. The fitness level for the measurement model shows a perfect fit. Assessing the fitness level for the measurement model for continuance commitment shows that the fitness level of the measurement model is achieved [Absolute fit (RMSEA) = .065, (ChiSq/df) = 4.468; Incremental fit (CFI) = .982; and Parsimonious fit (PNFI) = .520]. Hence, this study assumes that the unidimensionality for the measurement model for continuance commitment has been achieved. Therefore, no more modification was needed for this model.

### Assessing the measurement model for normative commitment

**Fig 5:**
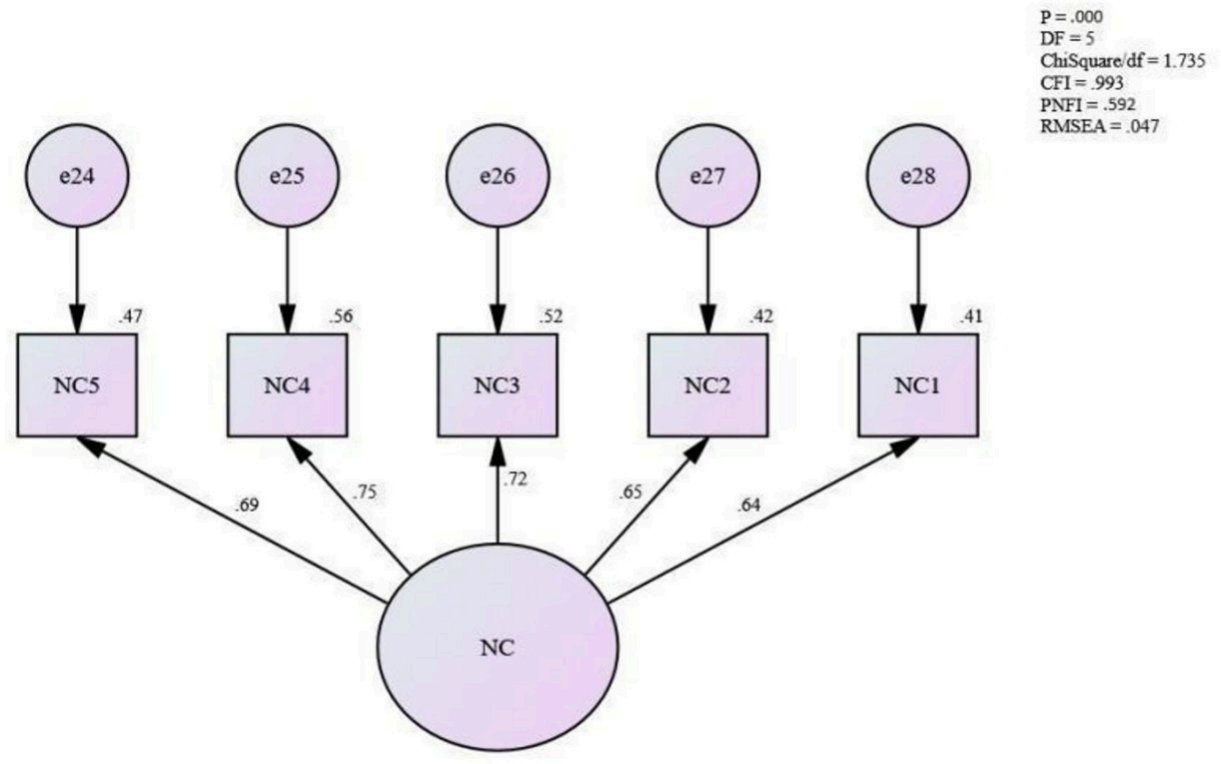
Measurement model for normative commitment.

Fig 5 shows the measurement model for normative commitment. It shows that the fitness level for the measurement model for normative commitment is achieved [Absolute fit (RMSEA) = .047, (ChiSq/df) = 1.735; Incremental fit (CFI) = .993; and Parsimonious fit (PNFI) = .592]. Hence, this study assumes that the unidimensionality for the measurement model for normative commitment has been achieved. Therefore, no more modification was needed for this model.

### Construct 3: Employee turnover intention

Identification of the dimensionality of employee turnover intention was explored through EFA and CFA. Three items were adapted to measure employee turnover intention. The item “For me, this company is the best of all possible organizations to work for” has achieved the highest mean average of 3.8906 (Table 10). This means that employees working in the public sector of the Sultanate of Oman are satisfied with their current jobs. On the other hand, the item “I am very likely to stay in this company for the next five years” has achieved the lowest average mean score of 3.245 (Table 10). This shows that majority of the employees working in the public sector of the Sultanate of Oman still prefer to work in their current organization though it scored the lowest mean average among other items. According to [20], a recent increase in pay in the public sector played a vital role in retaining the organisation’s current employees. The item identified in this study to measure employee turnover intention supports past studies [99, 102–105], which has defined employee turnover intention as any intention that an employee perceives or feels to leave the current organization for many reasons, such as unresolved gaps in the relationship between him/her and the organization. So, the psychological process is considered the most important reason that can affect employee attrition [58].

### Assessing the measurement model for employee turnover intention

**Fig 6:**
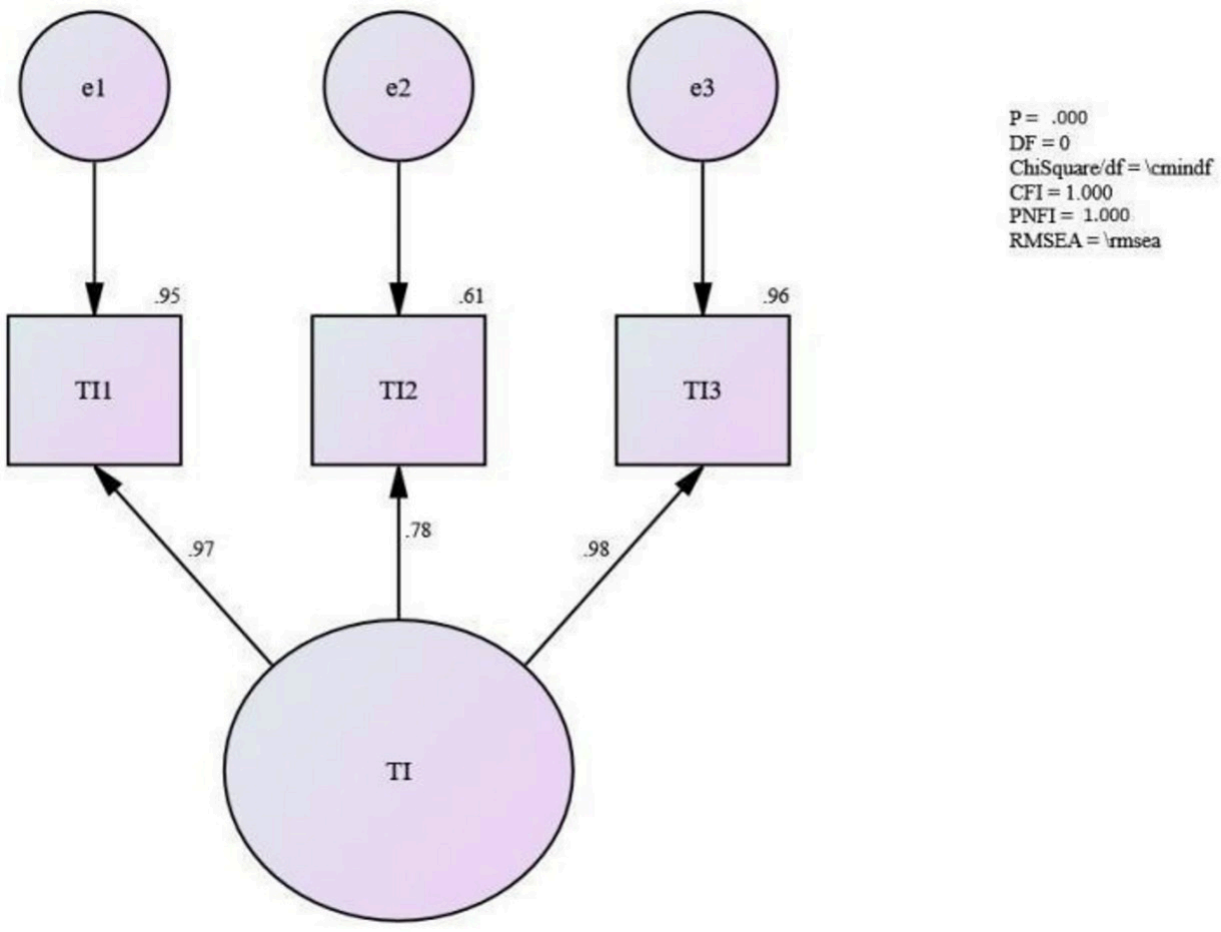
Measurement model for employee turnover intention.

From the fit of the measurement model (Fig 6), it can be seen that the required level for the absolute fit [Incremental fit (CFI) = 1.000; and Parsimonious fit (PNFI) = 1.000] was achieved. Hence, this study assumes that the unidimensionality for the measurement model for employee turnover intention has been achieved. However, according to [134], as the measurement model is left with only three items, the “df” value is always “zero”. As such, SEM software is unable to calculate the value for the absolute fit RMSEA and ChiSq/df since the model becomes a “just-identified” model. No further modification was needed for this model.

### Assessing the measurement model for all factors

**Fig 7:**
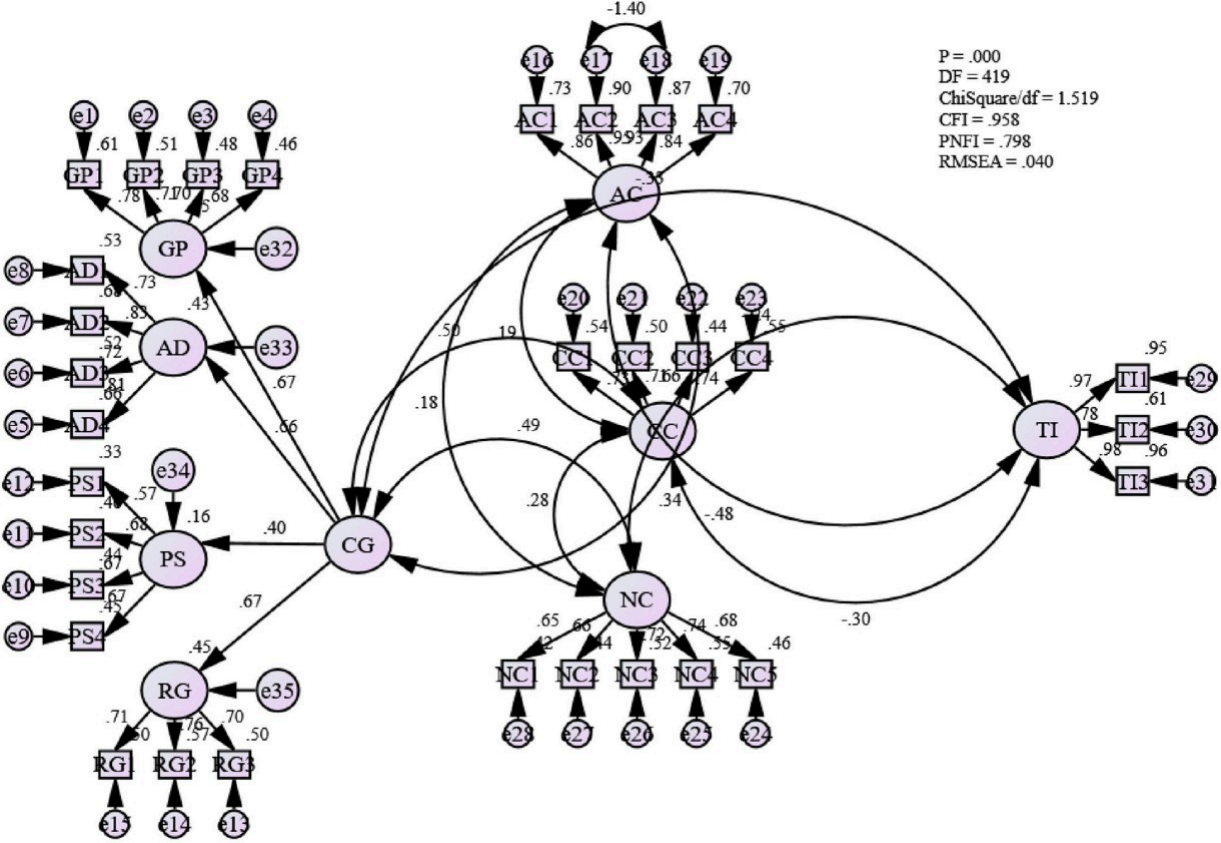
Measurement model for all factors.

The CFA analysis has extracted eight factors, Career goal progress (GP), Affective commitment (AC), Continuance Commitment (CC), Normative Commitment (NC), Remuneration Growth (RG), Professional Ability Development (AD) and Employee Turnover Intention (TI). From Fig 7, it can be observed that the fitness level for the measurement model has been achieved [Absolute fit (RMSEA) = .040, (ChiSq/df) = 1.519; Incremental fit (CFI) = .958; and Parsimonious fit (PNFI) = .798]. Hence, it was assumed that the model is fit for further analysis.

### Structural equation modelling for identifying the mediating role of organizational commitment

The Structural Equation Modelling (SEM) analyzes the interrelationships among the variables in a model [134]. Like other multivariate procedures, SEM also tests the theoretical model hypothesized by a researcher. In this stage, SEM was employed to identify the structural relationships between the different variables and their influences and to test hypotheses for this study. A series of goodness-of-fit indices that reflect the fitness of the model were used. Though there is no universal agreement among the researchers regarding which fitness indices should be used [120, 134] recommended using at least three fit indices, including at least one index from each category of fit model.

To assess the structural path relationships among the identified variables for this study, three distinct criteria have been applied based on [120]. The first criterion is the Absolute Fit category, where Root Mean Square Residuals (RMSEA) and ChiSq/df were used. The second category is the Incremental Fit, where the Comparative Fit Index (CFI) was considered. Finally, in the Parsimonious Fit category, this study has selected the Parsimony Normed Fit Index (PNFI). According to [138], in the case of the CFI, the closer the value is to 1, the better the fit of the model. In addition to this, [145] has mentioned that when the value is more than 0.90, it signifies an adequate fit to the data. This also ensures the incremental fit of the model. Besides, for RMSEA, a value less than 0.08 and ChiSq/df value less than three is well accepted [133, 135, 146] and signifies the absolute fit of the model. Finally, to achieve a parsimonious fit, and [135] suggested that the value of PNFI should be more than 0.50.

**Fig 8:**
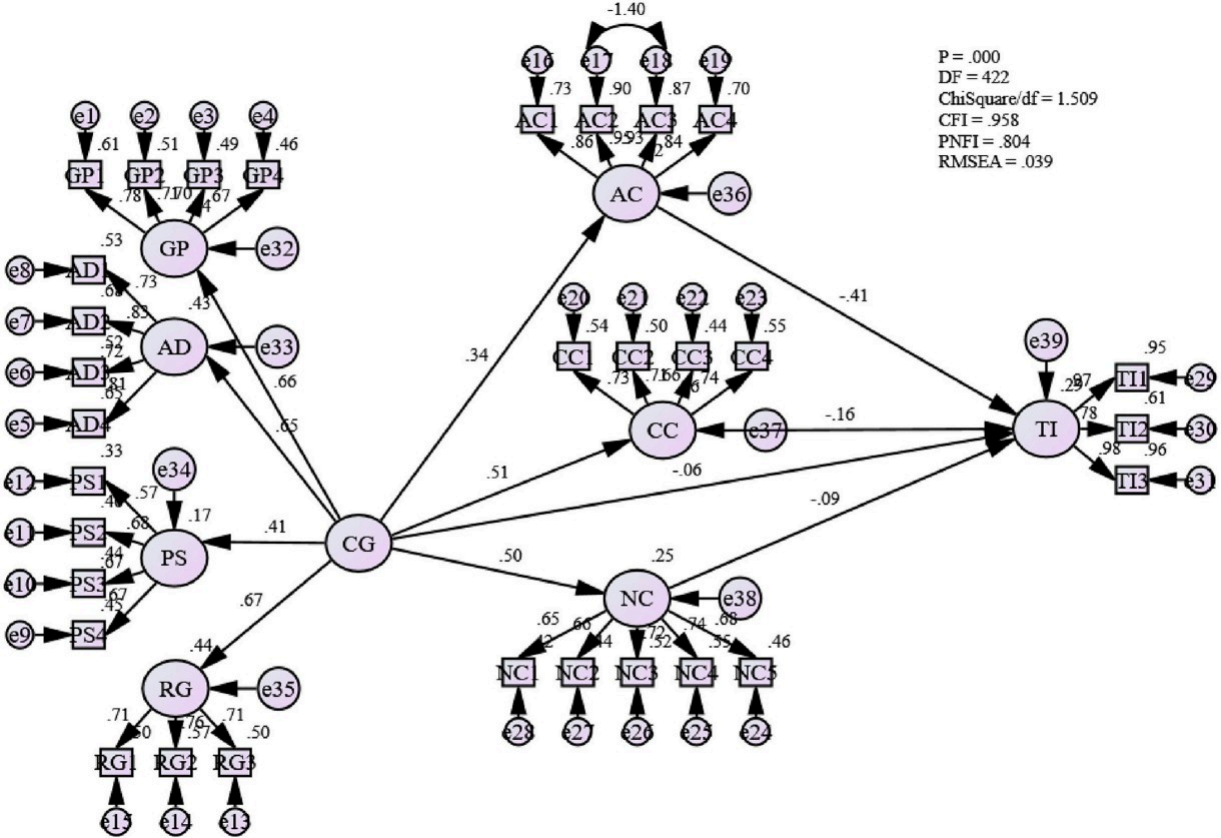
Fit indices and parameter estimates of hypothesized model.

Under this index, a proposed model has been compared with the null model, assuming that no relationship exists between the respected measures. Fig 8 illustrates the goodness-of-fit indices (GOF) values that have been attained from the SEM model for this study. It indicates that the fitness indices for the SEM model is achieved [Absolute fit (RMSEA) = .039, (ChiSq/df) = 1.509; Incremental fit (CFI) = .958; and Parsimonious fit (PNFI) = .804].

SEM is considered a meaningful statistical approach for its ability to disclose the existence of direct as well as indirect relationships between variables. In accordance with the above discussion, it can be perceived that employee turnover intention is heavily influenced by career goal progresses, professional ability development, promotion speed, remuneration growth, affective commitment, continuance commitment and normative commitment, as shown by the best fit model.

### Hypothesis testing

All the hypotheses of this study have been tested through the application of SEM. For the model as a whole, the statistical result indicates a good fit. The complete model is inclusive of the seven hypothesized paths, as illustrated in Table 11.

**Table 11:** Hypothesis testing.

		Estimate	SE	CR	P
Affective Commitment	Career Growth	0.344	0.095	3.621	***
Continuance Commitment	Career Growth	0.512	0.079	6.481	***
Normative Commitment	Career Growth	0.498	0.078	6.384	***
Turnover Intention	Career Growth	-0.063	0.111	-0.567	0.526

**Hypotheses: Organizational commitment and career growth–H1, H2 & H3**
**H1: There is a significant relationship between career growth and affective commitment in the Omani’ public sector**

**Fig 9:**
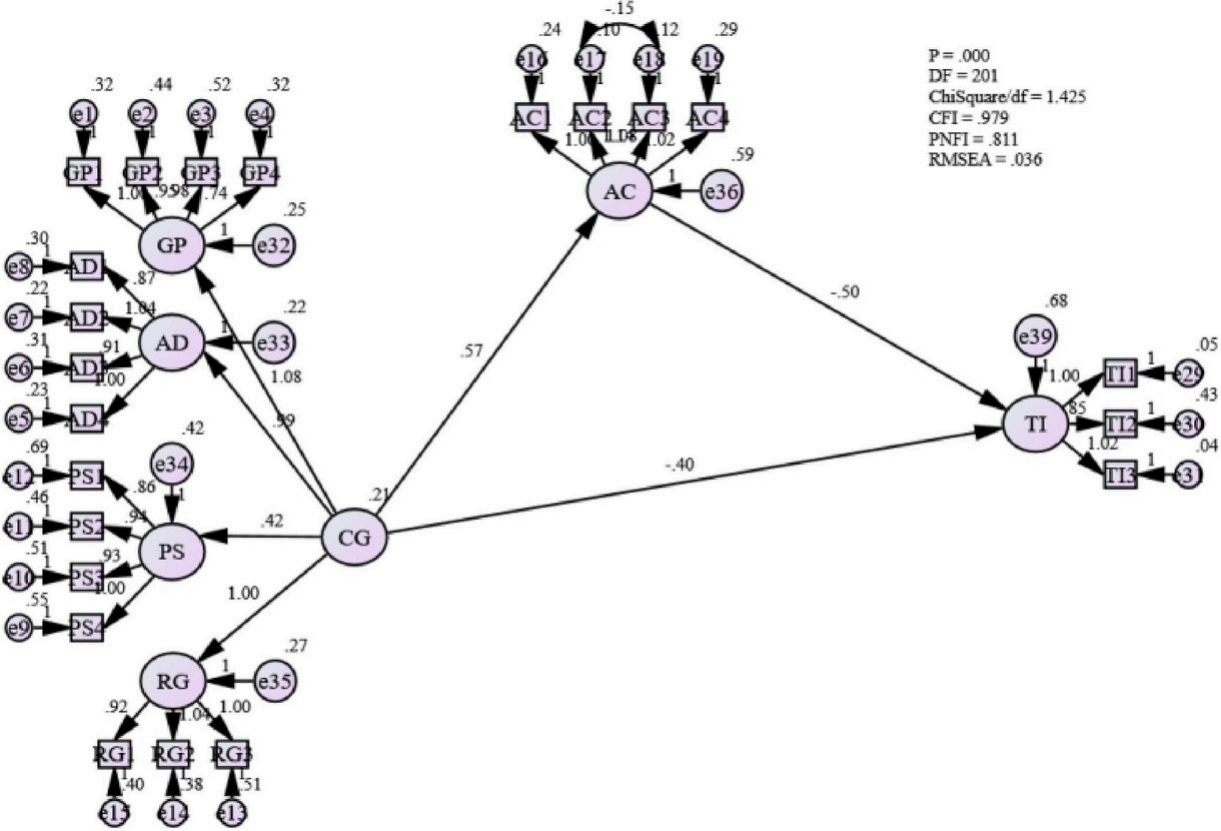
Mediating effect of affective commitment between career growth and turnover intentions.

This study employed SEM to identify the structural relationship between career growth and affective commitment in the Omani public sector. In Fig 9, it can be seen that the relationship between career growth and affective commitment in the Omani public sector is statistically significant with a path coefficient coefficient value of 0.344 (119). This also indicates that career growth significantly influences the affective commitment of employees in the Omani public sector.

Furthermore, from Table 11, it is also observed that the p-value is less than 0.05 (0.000) with a standard error of 0.095. Thus, this study accepts Hypothesis One that there is a positive relationship between career growth and affective commitment in the Omani public sector. This also answers the first research question: “What is the relationship between career growth and affective commitment in the Omani public sector?”. The finding is identical with past findings [100, 101]; where the authors have already pointed out that higher affective organizational commitment leads to a better job environment, as employees can feel the relationship between them and their employers show greater compatibility.

**H2: There is a significant relationship between career growth and continuance commitment in the Omani public sector**

In Table 10, the path coefficient between career growth and continuance commitment is positive 0.512. Thus, this study confirms that the relationship between career growth and continuance commitment in the Omani public sector is also statistically significant (the value of the path coefficient is more than 0.512). This indicates that the continuance commitment of employees in the Omani public sector significantly influences their career growth. Furthermore, from Table 11, it is also observed that the p-value is less than 0.05 (0.000) with a standard error of 0.079. Thus, this study accepts Hypothesis Two that there is a positive relationship between career growth and continuance commitment in the Omani public sector. This also answers the second research question: “What is the relationship between career growth and continuance commitment in the Omani public sector?”

The finding supports past findings [100, 102, 124, 129], where the authors pointed out that professional development opportunities are seen as an important determinant of employees’ behaviors and attitudes, which prevent obsolescence in their job skills. Thus, the increased promotional opportunities and pay raises can form continuance commitment.

**H3: There is a significant relationship between career growth and normative commitment in the Omani public sector**

In Table 10, it can be seen that the path coefficient between career growth and normative commitment is positive 0.498. Thus, this study confirms that the relationship between career growth and normative commitment in the Omani public sector is statistically significant. This also indicates that the normative commitment of employees in the Omani public sector significantly influences their career growth. Furthermore, from Table 10, it is also observed that the p-value is less than 0.05 (0.000) with a standard error of 0.078. Thus, this study accepts hypothesis three that there is a positive relationship between career growth and normative commitment in the Omani comparing public sector. This also answers research question three, which is “What is the relationship between career growth and normative commitment in the Omani public sector?”. The finding is also identical with past findings [1, 4, 98, 102–105, 147] where the authors have advocated that normative commitment plays an imperative role to retain employees as part of the organization, as it is a consequence of individual employee’s sense of obligation.

**Hypothesis: Turnover intention and career growth–H4**
**H4: Career growth is negatively associated with turnover intentions in the Omani public sector**

In Table 11, it can be seen that the path coefficient between career growth and turnover intentions in the Omani public sector is negative 0.063. Thus, this study confirms that the relationship between career growth and turnover intentions in the Omani public sector is negative. However, in this study, the relationship is not statistically significant (the value of the path coefficient is less than 0.15). Furthermore, from Table 11, it is also observed that the p-value is more than 0.05 (0.526) with a standard error of 0.111. Thus, this study rejects Hypothesis Four that career growth is significantly and negatively associated with turnover intentions in the Omani public sector. This study complements [53] research, which found that there is a negative association between turnover and promotion and salary growth. Thus, this study indicates that career growth does not significantly influence the turnover intentions of employees in the Omani public sector. Perhaps, the findings gave important lessons for HR practitioners in the Omani public sector, in that only providing opportunities for career growth might not help reduce attrition. What is pertinent is to develop ways and strategies in HRM that can boost organizational commitment, which subsequently lessens the intention to leave.

**Hypotheses: Organizational commitment and turnover intentions–H5, H6 & H7**
**H5: There is a significant negative relationship between affective commitment and turnover intention in the Omani public sector.**

In Table 12, it can be seen that the path coefficient between affective commitment and turnover intention is negative 0.414. Thus, this study confirms that the relationship between affective commitment and turnover intention in the Omani public sector is statistically significant. This also indicates that affective commitment negatively influences the turnover intentions of employees in the Omani public sector. Furthermore, from Table 10, it is also observed that the p-value is less than 0.05 (0.000) with a standard error of 0.054. Thus, this study accepts Hypothesis Five that there is a significant negative relationship between affective commitment and turnover intention in the Omani public sector. This also answers research question number V, which is “What is the relationship between affective commitment and employee’s intention to leave the public sector of the Sultanate of Oman?”. The finding is also identical with past findings [127–129], where the authors have reported that organizational commitment is connected closely to turnover intention. The employees’ intention for leaving the organization is considered based on their personal attachment to the organization. It is less likely for those who have a higher attachment to their organization.

**Table 12:** Hypothesis testing.

		Estimate	SE	CR	P
Turnover Intention	Affective Commitment	-0.414	0.054	-7.666	***
Turnover Intention	Continuance Commitment	-0.161	0.066	-2.439	0.021
Turnover Intention	Normative Commitment	-0.091	0.067	-1.358	0.081

**H6: There is a significant negative relationship between continuance commitment and turnover intention in the Omani public sector.**

In Table 12, it can be seen that the path coefficient between continuance commitment and turnover intention is negative 0.161. Thus, this study also confirms that the relationship between continuance commitment and turnover intention in the Omani public sector is statistically significant. This also indicates that affective commitment negatively influences the turnover intentions of employees in the Omani public sector. Furthermore, from Table 10, it is also observed that the p-value is less than 0.05 (0.000) with a standard error of 0.066. Thus, this study accepts Hypothesis Six that there is a significant negative relationship between continuance commitment and turnover intention in the Omani public sector. This also answers research question number 6, which is “What is the relationship between continuance commitment and employee’s intention to leave the public sector of the Sultanate of Oman?”.

Any intention that an employee perceives or feels towards attrition for many reasons, such as unresolved gaps in the relationship between him/her and the organization [27, 59]. So the psychological process is considered the most important reason that can cause employee attrition [58].

**H7: There is a significant negative relationship between normative commitment and turnover intention in the Omani public sector.**

In Table 12, it can be seen that the path coefficient between normative commitment and turnover intention is negative 0.091. Thus, this study confirms that the relationship between normative commitment and turnover intention in the Omani public sector is not statistically significant. However, this indicates that normative commitment negatively influences the turnover intentions of employees in the Omani public sector. Furthermore, from Table 12, it is also observed that the p-value is more than 0.05 (0.081) with a standard error of 0.067. Thus, this study rejects Hypothesis Seven that there is a significant negative relationship between normative commitment and turnover intention in Omani’s public sector. This also answers research question number 7, which is “What is the relationship between normative commitment and employee’s intention to leave the public sector of the Sultanate of Oman?”.

According to past authors [84, 87, 99]; employees may achieve excellent performance when they are committed to their organization. Additionally, [148] found that employees can give discretionary effort as well as have a greater impact on the performance and effectiveness of organizational strategies if they have been motivated as skilled and knowledgeable people to achieve their tasks. Normative commitment as an obligation may not induce greater effort voluntarily but perhaps may contribute to inertia and indifference to the organization. This essentially is not normative commitment. Interestingly [20] suggest that cultural factors may contribute to inertial effects, as they discovered normative commitment or sense of obligation owing to Islamic values in Omani organizations. A past study conducted by [103] has found that normative commitment is an obligation deriving from the personality of the employee and the personal value resulting from that feeling of obligation to remain in the organization. Hence, it refers to a mindset of obligation rather than the mindset of desire [105]. Normative commitment plays a role to keep employees in the same organization due to their own sense of obligation [125]. In other words, receiving benefits was perceived by employees as a mutually obligatory expectation that will lead to normative commitment.

**Hypotheses: The mediating role of organizational commitment–H8, H9 & H10**
**H8: Affective Commitment Mediates the Relationship between Career Growth and Turnover Intentions**

**Fig 10:**
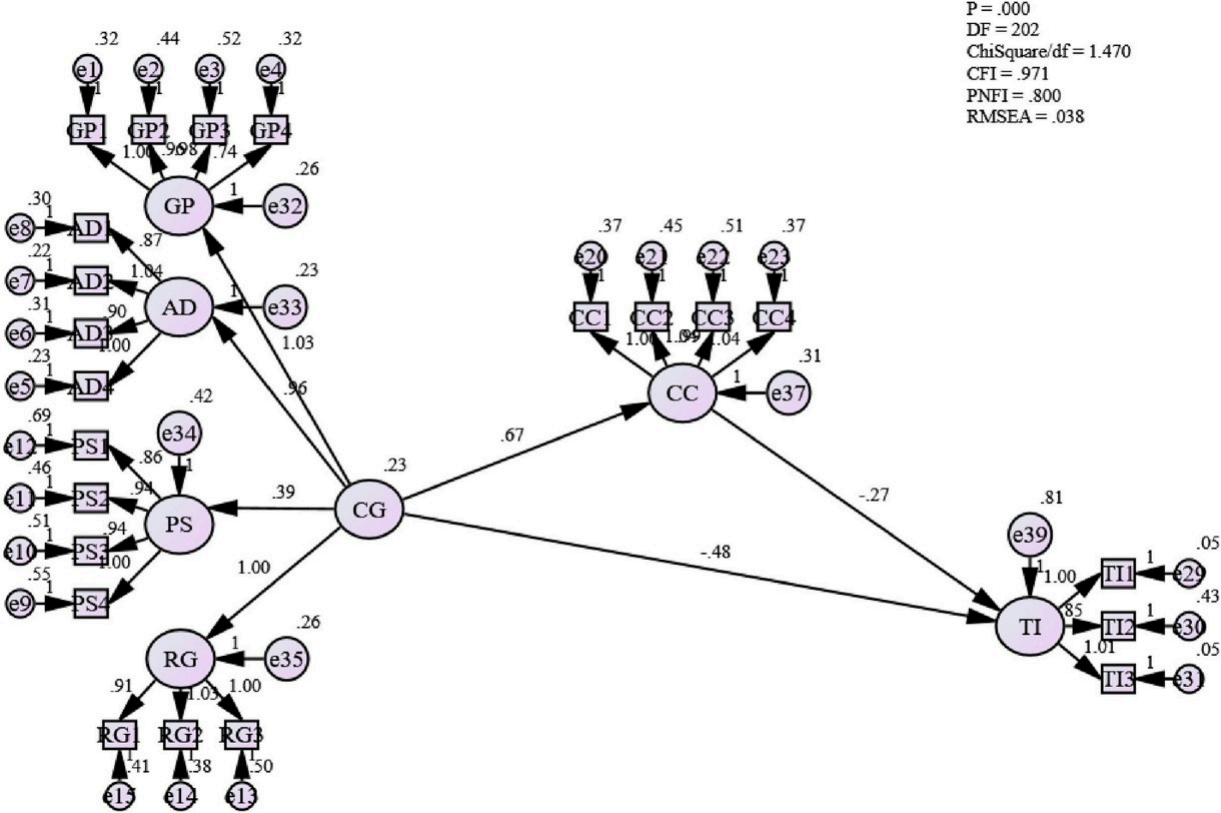
Mediating effect of continuance commitment between career growth and turnover intentions.

This study has confirmed that organizational commitment (affective and continuance, except normative commitment) mediated the relationship between career growth and employee turnover intention. Career growth was found to be an insignificant predictor of employee turnover intention, but when Organizational commitment was included in the equation as a mediator, career growth became significant. This indicated that career growth’s role is pivotal in relation to organizational commitment. Therefore, the positive relationship between career growth and turnover intention infers that when employees are given sufficient support in their personal development and growth, they perceive themselves as more marketable and employable in other organizations. Fig 10 shows the mediating effect of affective commitment between career growth and turnover intentions. It explains the goodness-of-fit indices (GOF) values that have been attained from the model for this study. It indicates that the fitness indices for the SEM model have been achieved [Absolute fit (RMSEA) = .036, (ChiSq/df) = 1.425; Incremental fit (CFI) = .979; and Parsimonious fit (PNFI) = .811]. According to [133], if all the relationships are statistically significant, then it can be assumed that there is a partial mediation. However, if the direct relationship becomes insignificant when mediators are included in the model, then it signifies a full mediation.

In this case, to confirm the types of mediation (full or partial), this study has tested the relationship presented in Fig 9 and Table 13. It can be observed that the relationship between affective commitment and career growth is statistically significant with a path value of positive 0.57. The relationship between career growth and turnover intention is also statistically significant, with a path value of negative 0.40. The relationship between affective commitment and turnover intention is also statistically significant, with a path value of negative .50. Hence, this indicates a partial mediation in the model. This also answers the research question, “Whether affective commitment mediates between career growth and turnover intention?”.

**Table 13:** Regression weights.

		Estimate	SE	CR	p
Affective Commitment	Career Growth	0.571	0.137	4.171	***
Turnover Intention	Career Growth	-0.396	0.150	-2.634	0.008
Turnover Intention	Affective Commitment	-0.497	0.065	-7.656	***

Past studies have discussed that affective commitment is the psychological attachment of employees to their organization that emphasizes their identification with their organization’s objectives and values [122]. [130] found that affective commitment has a negative impact on turnover intention. [8] has found that career growth has a positive impact on affective commitment. Thus, this study accepts Hypothesis Eight that affective commitment mediates the relationship between career growth and turnover intentions.

**H9: Continuance Commitment Mediates the Relationship between Career Growth and Turnover Intentions**

**Fig 11:**
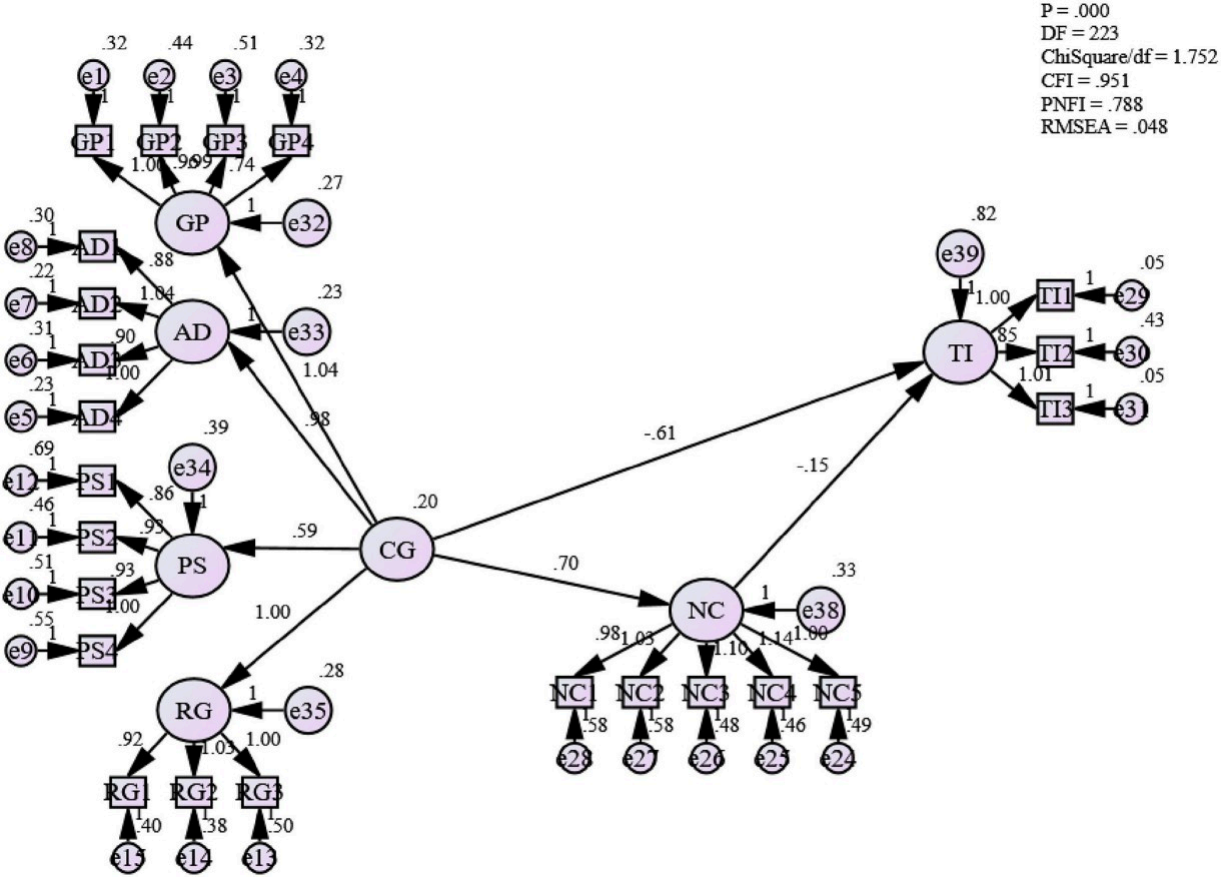
Mediating effect of normative commitment between career growth and turnover intentions.

Fig 11 shows the mediating effect of continuance commitment between career growth and turnover intentions. It explains the goodness-of-fit indices (GOF) values that have been attained from the model for this study. It indicates that the fitness indices for the SEM model has achieved [Absolute fit (RMSEA) = .038, (ChiSq/df) = 1.470; Incremental fit (CFI) = .971; and Parsimonious fit (PNFI) = .800].

To confirm the types of mediation (full or partial mediation), this study has tested the relationship presented in Fig 10 and Table 14. It can be observed that the relationship between continuance commitment and career growth is statistically significant with a path value of positive .67. The relationship between career growth and turnover intention is also statistically significant, with a negative path value of .27. The relationship between continuance commitment and turnover intention is also statistically significant, with a path value of negative .48. Hence, this also indicates a partial mediation in the model. This also answers research question number nine, which is “Does continuance commitment mediate between career growth and turnover intention?”.

**Table 14:** Regression weights.

		Estimate	SE	CR	p
Continuance Commitment	Career Growth	0.673	0.126	5.328	***
Turnover Intention	Career Growth	-0.477	0.183	-2.614	0.009
Turnover Intention	Continuance Commitment	-0.270	0.113	-2.382	0.017

The findings are also similar to past findings. Continuance commitment is about the employees’ belief that they will lose benefits or seniority status [149]. According to [150], employees in their current organization have gained many things, such as training and development, reducing attrition. A study conducted by [39] has found a positive relationship between continuance commitment and career growth. However, [151] found that continuance commitment is negatively associated with employee turnover intention. Thus, this study accepts Hypothesis Nine that continuance commitment mediates the relationship between career growth and turnover intention.

**H10: Normative Commitment Mediates the Relationship between Career Growth and Turnover Intentions**

Fig 11 shows the mediating effect of normative commitment between career growth and turnover intentions. It explains the goodness-of-fit indices (GOF) values that have been attained from the model for this study. It indicates that the fitness indices for the SEM model have been achieved [Absolute fit (RMSEA) = .048, (ChiSq/df) = 1.752; Incremental fit (CFI) = .951; and Parsimonious fit (PNFI) = .788].

To confirm the types of mediation (full or partial mediation), this study has tested the relationship presented in Fig 11 and Table 15. It can be observed that the relationship between normative commitment and career growth is statistically significant, with a path value of positive .70. The relationship between career growth and turnover intention is also statistically significant, with a negative path value of .61. However, the relationship between normative commitment and turnover intention is not statistically significant, with a negative path value of .15 (p ≥ 0.05). Hence, this indicates that normative commitment does not mediate the relationship between career growth and turnover intention in the model. This also answers research question number ten which is “Does normative commitment mediate between career growth and turnover intention?”.

**Table 15:** Regression weights.

		Estimate	SE	CR	p
Normative Commitment	Career Growth	0.699	0.150	4.661	***
Turnover Intention	Career Growth	-0.607	0.200	-3.032	0.002
Turnover Intention	Normative Commitment	-0.150	0.112	-1.332	0.183

In the past, it was found that career growth is related to employees’ behaviors and attitudes that can bring about turnover intention if career development is not managed accurately. [26] explained that career growth is negatively correlated to turnover intention. Furthermore, [126, 130] found that normative commitment negatively affects turnover intention rather than career growth. Meyer further supported these findings, as well as [103], who pointed out that normative commitment is the mindset of obligation rather than the mindset of desire. Thus, this study rejects Hypothesis 10 that normative commitment mediates the relationship between career growth and turnover intentions.

## 4. Conclusion and recommendations

This study at hand aimed at exploring the relationship between career growth and organizational commitment, and whether, affective commitment, continuance commitment, and normative commitment mediates the relationship between career growth and employee turnover intention among the employees working in the public sector of the Sultanate of Oman. The results indicate that career growth is very essential element that motivates employees’ turnover intention in the public sector of the Sultanate of Oman. In addition, the findings also confirm that affective and continuance except normative commitment significantly mediates the relationship between career growth and employee turnover intention. According to this study, the public sector in Oman failed to retain talented employees due to two main interconnected reasons: the lack of a clear career program by the employers that lead to the lack of strong organizational commitment among employees. This study provided significant insights in identifying career growth by utilizing the four components as a second-order that influence employee turnover intention in the public sector of the Sultanate of Oman. Furthermore, this study has also identified the key dimensions of organizational commitment (e.g. affective, continuance and normative commitment) that mediate the relationship between career growth and employee turnover intention. The findings of this research have confirmed that affective and continuance commitment are the key factors that mediate career growth and employees’ turnover intention relationship in the public sector of the Sultanate of Oman context. However, the findings on the relationship between career growth and turnover intentions and the relationship between normative commitment and turnover intention were found to be statistically not significant (p>.05), specifically from the Omani perspective. This finding should be taken seriously by managers because employees who have the low commitment and thus high turnover intention are not likely to feel compelled to commit and give all their efforts to the organizations. Any new building of career growth of the organization’s employees or any initiative that aims to retain employees must first address the need to increase employees’ commitment to the organization.

### Theoretical implications

This study is theoretically significant for the field of Organizational Behavior (OB) as it has contributed to a theoretical enhancement of the current level of knowledge in the existing literature on OB. The mediating role of organizational between career growth and turnover intention were considered an extension of what [81] have stated. [81] examined affective commitment as a single component of organizational commitment. Consequently, this creates the necessity for a complete examination of all organizational commitment components. This study filled this gap by adding two more variables in organizational commitment, namely continuance commitment and normative commitment; which have been accounted as a major contribution to organizational commitment.

### Methodological implications

This study has proposed a measurement tool through which career growth and organizational commitment and turnover intention can be measured. This study provides a valid and reliable instrument that can measure the relationship between career growth, organizational commitment and turnover intention. This could contribute significantly to the methodological development for HRM research. In addition, this study could help explore the organizational and individual factors to shed more light on what individuals and organizations can do to facilitate the growth of careers. The results of this analysis will help organizations to learn from the survey-based case study and apply major findings from the study into their own settings and hence improve their employee relations practices and provide insights for future growth in the field.

### Empirical implications

Empirically, this research seeks to evaluate the mediating role of organizational commitment (affective, continuance and normative commitment) between the relationship career growth (career goal progresses, professional ability development, promotion speed and remuneration growth) as sub-construct and employee turnover intention. Firstly, a total of five variables were identified in this study, they include one independent variable (career growth), three mediating variables (affective, continuance and normative commitment) as well as one dependent variable (employee turnover intention). Secondly, this study has significantly contributed to the development of HRM theory on career growth, organizational commitment and turnover intention. This study was undertaking empirical testing of the structural relationships among the study factors. This systematic examination of structural relationships among the constructs has facilitated a clearer understanding of the nature of the employee turnover intention from the career growth and organizational commitment view.

### Practical implications

This research has practical significance as currently a very limited number of studies have been conducted on the issue specifically focused on the public sector of the Sultanate of Oman (e.g. [108, 110]). Current research enables a superior understanding of the factors behind the turnover intention that the public administration in the Gulf countries in general and Omani public administration faces in particular. This study will help achieve the overall research of turnover goals, and the result may be generalized to similar countries. Therefore, its resulting indications could help both researchers and those involved in public sector policy to understand how employee turnover intention is influenced by career growth and organizational commitment in the public sector of the Sultanate of Oman. Besides, this research finding will contribute to further development of the Oman public sector HRM policy and those associated with it.

Thus, to conclude, this research expects to create a working awareness of the factors that promote or deter career growth and organizational commitment among individuals and organizations in the public sector in Oman. The results from this research will help practitioners to manage human resources and appreciate individual differences factors in career growth and organizational commitment to reduce turnover. Managers in the Omani public sector must take these findings seriously because employees who have a low commitment and high turnover intention are not likely to feel compelled to commit and give all their efforts to the organizations. The most important conclusion of this research is that while building career growth programs aimed at increasing the employee retention, key factor that needs to be addressed is the employees’ commitment to the organization.

### Limitations of the study and future direction

In this study, the data collection was challenging as the respondents were employees working in the Omani public sector. As such, accessing the right respondents was very challenging. Furthermore, identifying the right respondent was also very difficult as this study only considered those employees with three or more years of working experience. Hence, the researchers had to ask every single respondent who was available during the data collection, whether they have more than three years of work experiences or not. Nevertheless, the empirical model was found to have a good fit based on the data gathered from the respondents.

As information regarding the current HRM practices in Oman is not readily available, Omani public organizations need to publicize their success stories on their websites or in the newspapers, helping them to find out the best practices in the coming future. Besides, this study also proposes to replicate the process into the private sector in the future and see whether a significant difference exists between the public and private sectors. Finally, an investigation of the impact of employees’ demographic variables such as gender, age, job title and education level as mediating variables is likely to provide a better understanding of the relationship. The scopes of unexplained variance indicate that factors not included in the framework of the study might be important to consider.

### Contribution and originality value of the study

This study aims to extend the relationship between career growth and turnover intention by using model proposed by [26]. This model has proposed four main constructs of Organizational Career growth, viz., career goal progress, professional ability development, promotion speed and remuneration growth. However, in this study, career growth in the Omani public sector was explored by examining the relationship between the dependent variable of turnover intention via a mediating viable of commitment that could lead to fuller rational explanations of their inherent connections practically among the public sector’s employees in the Sultanate of Oman. Therefore, this field study is an applied extension from actual organizational field data from theoretical, methodological and actual field research standpoint.

The main purpose of this study was to examine organizational commitment (affective, continuance and normative based on Meyer and Allen’s 1991 model) as mediating variables between career growth and turnover intention in the Omani public sector. In the process, firstly, researchers placed turnover intention as a dependent variable by using career growth directly as an independent variable and also organizational commitment components as independents (affective, normative and continuance commitment). Secondly, mediating organizational commitment components, it enabled a superior understanding of the factors behind the turnover intention that the public administration, in the Gulf countries in general and in Omani public administration in particular, actually faces. In addition, [20] conducted a qualitative study in Oman, and it appears that organizational commitment is a major theme that has indicated diminished normative commitment. Consequently, this reinforces the need for further exploratory testing of all three components of organizational commitment quantitatively.

In this context, this study helps to achieve the overall research of the causes of turnover, and the result may be generalizable to similar countries. Therefore, its resulting indications could help both researchers and those involved in public sector policy to understand how employee’s turnover intention is influenced by career growth and organizational commitment in the public sector of the Sultanate of Oman.

## Supporting information

S1 Appendix Mahalanobis distance (Observations Farthest from the Centroid). (DOCX)

## References

[1] Chun-Yu L. and Chung-Kai H., “Employee turnover intentions and job performance from a planned change: the effects of an organizational learning culture and job satisfaction,” *International Journal of Manpower*, vol. ahead-of-p, no. ahead-of-print. pp. 1–15, Jan. 01, 2020, doi: 10.1108/IJM-08-2018-0281

[2] Zingheim P. K., Ph D., and Schuster J. R., “Managing Total Compensation To Achieve Multiple Objectives,” vol. 7, no. 1, pp. 37–40, 2008.

[3] Curristine T., Lonti Z., and Joumard I., “Improving Public Sector Efficiency: Challenges and Opportunities,” *OECD J*. *Budg*., vol. 7, no. 1, pp. 1–41, 2007, doi: 10.1787/budget-v7-art6-en

[4] Brunetto Y. and Beattie R., “Changing role of HRM in the public sector,” *Public Manag*. *Rev*., vol. 22, no. 1, pp. 1–5, 2020, doi: 10.1080/14719037.2019.1645876

[5] Ali A. M., “Demographic factors, compensation, job satisfaction and organizational commitment in private university: an analysis using SEM,” *J*. *Glob*. *Responsib*., vol. 11, no. 4, pp. 407–436, Jan. 2020, doi: 10.1108/JGR-01-2020-0010

[6] Allen D. G., Shore L. M., and Griffeth R. W., “The Role of Perceived Organizational Support and Supportive Human Resource Practices in the Turnover Process,” *J*. *Manage*., vol. 29, no. 1, pp. 99–118, 2003, doi: 10.1177/014920630302900107

[7] M. A. and Andy Soenanta R. T. S., “The effect of job satisfaction and organizational commitment to employee retention in a lighting company,” *Issues Bus*. *Manag*. *Econ*., vol. 8, no. 4, pp. 97–103, 2020.

[8] Weng Q. and Zhu L., “Individuals’ Career Growth Within and Across Organizations: A Review and Agenda for Future Research,” *J*. *Career Dev*., vol. 47, no. 3, pp. 239–248, 2020, doi: 10.1177/0894845320921951

[9] Younas M. and Bari M. W., “The relationship between talent management practices and retention of generation ‘Y’ employees: mediating role of competency development,” *Econ*. *Res*. *Istraz*., vol. 33, no. 1, pp. 1330–1353, 2020, doi: 10.1080/1331677X.2020.1748510

[10] Junaid Khan A. and Iqbal J., “Training and Employee Commitment: The Social Exchange Perspective,” *J*. *Manag*. *Sci*., vol. 7, no. 1, pp. 88–100, 2020, doi: 10.20547/jms.2014.2007106

[11] Buchenroth P., “Driving Performance: Making Pay Work for the Organization,” *Compens*. *Benefits Rev*., vol. 38, no. 3, pp. 30–35, 2006, doi: 10.1177/0886368706288210

[12] Huo W., Li X., Zheng M., Liu Y., and Yan J., “Commitment to human resource management of the top management team for green creativity,” *Sustain*., vol. 12, no. 3, 2020, doi: 10.3390/su12031008

[13] Chew Y. T., “Achieving Organisational Prosperity through Employee Motivation and Retention: A Comparative Study of Strategic HRM Practices in,” *Res*. *Pract*. *Hum*. *Resour*. *Manag*., vol. 13, no. 2, pp. 87–104, 2005.

[14] Hofstede G., “Motivation, Leadership, and Organization: Do American Theories Apply Abroad?,” *Am*. *Manag*. *Assoc*., pp. 42–63, 1980, [Online]. Available: https://pdf.sciencedirectassets.com/272151/1-s2.0-S0090261600X01088/1-s2.0-0090261680900133/main.pdf?X-Amz-Security-Token=IQoJb3JpZ2luX2VjEB8aCXVzLWVhc3QtMSJHMEUCIGJMyj4W6enrOFcvS0MZePqLPBWwDcGqy7Ygk3ww1E9MAiEAn3DwzNF6F8VYD9L9dTP3AeXUZwyKzhX13p3FHSCKT9Mqv

[15] Liu Y., Liu J., and Wu L., “Are you willing and able? Roles of motivation, power, and politics in career growth,” *J*. *Manage*., vol. 36, no. 6, pp. 1432–1460, 2010, doi: 10.1177/0149206309359810

[16] Thomas K. W., *Intrinsic Motivation at Work*: *What Really Drives Employee Engagement*, Second Edi. Berrett-Koehler Publishers, 2009.

[17] Omanuna, “Entities List,” *Entities List*, pp. 1–14, 2021.

[18] Al Sherbini R., “Indians top foreign workers in Oman ‘ s public sector,” *Indians top foreign workers in Oman’s public sector*, pp. 1–6, 2021.

[19] Al Kurdi B., Alshurideh M., and Al afaishat T., “Employee retention and organizational performance: Evidence from banking industry,” *Manag*. *Sci*. *Lett*., vol. 10, no. 16, pp. 3981–3990, 2020, doi: 10.5267/j.msl.2020.7.011

[20] Swailes S. and Al Fahdi S., “Voluntary turnover in the Omani public sector: An islamic values perspective,” *Int*. *J*. *Public Adm*., vol. 34, no. 10, pp. 682–692, 2011, doi: 10.1080/01900692.2011.583770

[21] Biswakarma G., “International Academic Journal of Organizational Behavior and Human Resource Management Organizational Career Growth and Employees " Turnover Intentions: An empirical evidence from Nepalese Private Commercial Banks,” Int. Acad. J. *Organ*. *Behav*. *Hum*. *Resour*. *Manag*., vol. 3, no. 2, pp. 10–26, 2016, [Online]. Available: www.iaiest.com

[22] Arthur M. B., “Examining contemporary careers: A call for interdisciplinary inquiry,” *Hum*. *Relations*, vol. 61, no. 2, pp. 163–186, 2008, doi: 10.1177/0018726707087783

[23] Hirschi A., Zacher H., and Shockley K. M., “Whole-Life Career Self-Management: A Conceptual Framework,” *J*. *Career Dev*., no. June, 2020, doi: 10.1177/0894845319832972 32742075 PMC7394468

[24] Sturges J., Guest D., Conway N., and Davey K. M., “What Difference Does It Make? a Longitudinal Study of the Relationship Between Career Management and Organizational Commitment in the Early Years At Work.,” *Acad*. *Manag*. *Proc*., vol. 2001, no. 1, pp. B1–B6, 2001, doi: 10.5465/apbpp.2001.6132958

[25] Luedech Girdwichai C. S., “EMPLOYEE MOTIVATION AND PERFORMANCE: DO THE WORK ENVIRONMENT AND THE TRAINING MATTER?,” *J*. *Secur*. *Sustain*. ISSUES, vol. 9, no. 8, pp. 96–107, 2020.

[26] Weng Q., McElroy J. C., Morrow P. C., and Liu R., “The relationship between career growth and organizational commitment,” *J*. *Vocat*. *Behav*., vol. 77, no. 3, pp. 391–400, 2010, doi: 10.1016/j.jvb.2010.05.003

[27] Karavardar G., “Organizational Career Growth and Turnover Intention: An Application in Audit Firms in Turkey,” *Int*. *Bus*. *Res*., vol. 7, no. 9, pp. 67–76, 2014, doi: 10.5539/ibr.v7n9p67

[28] Guterman M., “Career growth: a model and methods for changing times,” *Int*. *J*. *Career Manag*., vol. 3, no. 1, pp. 3–7, 1991.

[29] Okurame D., “Impact of career growth prospects and formal mentoring on organisational citizenship behaviour[,” *Leadersh*. *Organ*. *Dev*. *J*., vol. 33, no. 1, pp. 66–85, 2012.

[30] Tabachnick B. G. and Fidell L. S., *Using multivariate statistics*, 5th ed. Boston, MA: Allyn & Bacon/Pearson Education, 2007.

[31] Cohen A., “Commitment before and after: An evaluation and reconceptualization of organizational commitment,” *Hum*. *Resour*. *Manag*. *Rev*., vol. 17, no. 3, pp. 336–354, 2007, doi: 10.1016/j.hrmr.2007.05.001

[32] Davidescu A. A. M., Apostu S. A., Paul A., and Casuneanu I., “Work Flexibility, Job Satisfaction, and Job Performance among Romanian Employees—Implications for Sustainable Human Resource Management,” *Sustain*., vol. 12, no. 15, pp. 1–53, 2020, doi: 10.3390/su12156086

[33] Choi S., “Organizational justice and employee work attitudes: The federal case,” *Am*. *Rev*. *Public Adm*., vol. 41, no. 2, pp. 185–204, 2011, doi: 10.1177/0275074010373275

[34] Park S. and Choi S., “Performance feedback, goal clarity, and public employees’ performance in public organizations,” *Sustain*., vol. 12, no. 7, pp. 1–18, 2020, doi: 10.3390/su12073011

[35] Erdogan B., Liden R. C., and Kraimer M. L., “Justice and leader-member exchange: The moderating role of organizational culture,” *Acad*. *Manag*. *J*., vol. 49, no. 2, pp. 395–406, 2006, doi: 10.5465/AMJ.2006.20786086

[36] Ho W. H., Chang C. S., Shih Y. L., and Da Liang R., “Effects of job rotation and role stress among nurses on job satisfaction and organizational commitment,” *BMC Health Serv*. *Res*., vol. 9, no. 1, pp. 1–10, 2009, doi: 10.1186/1472-6963-9-8 19138390 PMC2630925

[37] James K., Robert Jp., and Terry S., “Career-Related Benefits and Turnover Intentions in Accounting Firms: The Roles of Career Growth Opportunities, Trust in Superiors, and Organizational Commitment,” in *Advances in Accounting Behavioral Research*, vol. 20, Emerald Publishing Limited, 2017, pp. 1–21.

[38] Horn P. W. et al., “Structural Equations Modeling Test of a Turnover Theory—Cross-Sectional and Longitudinal Analyses,” *J*. *Appied Psychol*., vol. 76, no. 3, pp. 350–366, 1991.

[39] Gong Y., Law K. S., Chang S., and Xin K. R., “Human Resources Management and Firm Performance: The Differential Role of Managerial Affective and Continuance Commitment,” *J*. *Appl*. *Psychol*., vol. 94, no. 1, pp. 263–275, 2009, doi: 10.1037/a0013116 19186911

[40] Kim S. W., Price J. L., Mueller C. W., and Watson T. W., “The determinants of career intent among physicians at a U.S. Air Force hospital,” *Hum*. *Relations*, vol. 49, no. 7, pp. 947–976, 1996, doi: 10.1177/001872679604900704

[41] Lankau M. J. and Scandura T. A., “An investigation of personal learning in mentoring relationships: Content, antecedents, and consequences,” *Acad*. *Manag*. *J*., vol. 45, no. 4, pp. 779–790, 2002, doi: 10.2307/3069311

[42] Miao Q., Newman A., Sun Y., and Xu L., “What factors influence the organizational commitment of public sector employees in China? The role of extrinsic, intrinsic and social rewards,” *Int*. *J*. *Hum*. *Resour*. *Manag*., vol. 24, no. 17, pp. 3262–3280, 2013, doi: 10.1080/09585192.2013.770783

[43] Nordhaug O., “Reward Functions of Personnel Training,” *Hum*. *Relations*, vol. 42, no. 5, pp. 373–388, 1989, doi: 10.1177/001872678904200501

[44] Nouri H. and Parker R. J., “Career growth opportunities and employee turnover intentions in public accounting firms,” *Br*. *Account*. *Rev*., vol. 45, no. 2, pp. 138–148, 2013.

[45] Luna-Arocas R. and Lara F. J., “Talent management, affective organizational commitment and service performance in local government,” *Int*. *J*. *Environ*. *Res*. *Public Health*, vol. 17, no. 13, pp. 1–15, 2020, doi: 10.3390/ijerph17134827 32635534 PMC7369947

[46] Kyle Ehrhardt P. W. H., Miller Janice S., Freeman Sarah J., “An Examination of the Relationship Between Training Comprehensiveness and Organizational Commitment: Further Exploration of Training Perceptions and Employee Attitudes,” *Comput*. *Complex*., vol. 22, no. 4, pp. 459–489, 2011, doi: 10.1002/hrdq

[47] Ng T. W. H., Butts M. M., Vandenberg R. J., DeJoy D. M., and Wilson M. G., “Effects of management communication, opportunity for learning, and work schedule flexibility on organizational commitment,” *J*. *Vocat*. *Behav*., vol. 68, no. 3, pp. 474–489, 2006, doi: 10.1016/j.jvb.2005.10.004

[48] Robianto F., Masdupi E., and Syahrizal, “The Effect of Career Development, Compensation, Work Environment and Job Satisfaction on Work Engagement,” vol. 124, pp. 737–748, 2020, doi: 10.2991/aebmr.k.200305.140

[49] Dabos G. E. and Rousseau D. M., “Mutuality and Reciprocity in the Psychological Contracts of Employees and Employers,” *J*. *Appl*. *Psychol*., vol. 89, no. 1, pp. 52–72, 2004, doi: 10.1037/0021-9010.89.1.52 14769120

[50] Nicky D., Roland P., and Evelien D. K., “Exploring four generations’ beliefs about career: Is ‘satisfied’ the new ‘successful’?,” *J*. *Manag*. *Psychol*., vol. 23, no. 8, pp. 907–928, Jan. 2008, doi: 10.1108/02683940810904394

[51] Seibert S. E., Kraimer M. L., and Liden R. C., “A social capital theory of career success,” *Acad*. *Manag*. *J*., vol. 44, no. 2, pp. 219–237, 2001, doi: 10.2307/3069452

[52] Giraud L., Bernard A., and Trinchera L., Early career values and individual factors of objective career success: The case of the French business graduates, vol. 24, no. 4. 2019.

[53] Salamin A. and Hom P. W., “In Search of the Elusive U-Shaped Performance-Turnover Relationship: Are High Performing Swiss Bankers More Liable to Quit?,” *Journal of Applied Psychology*, vol. 90, no. 6. American Psychological Association, Salamin, Alain: Ecole hoteliere de Lausanne, Le Chalet-a-Gobet, Lausanne, Switzerland, 1000, 25, alain.salamin@ehl.ch, pp. 1204–1216, 2005, doi: 10.1037/0021-9010.90.6.1204 16316274

[54] Blau P. M., *Exchange and power in social life*. New York: J. Wiley, 1964.

[55] Rousseau D. M., “Psychological and implied contracts in organizations,” *Empl*. *Responsib*. *Rights J*., vol. 2, no. 2, pp. 121–139, 1989, doi: 10.1007/BF01384942

[56] Malhotra N., Budhwar P., and Prowse P., Linking rewards to commitment: An empirical investigation of four UK call centres, vol. 18, no. 12. 2007.

[57] Cho W. S., Kim S., Han B. S., Son W. C., and Jeong J., “Comparison of gene expression profiles in mice liver following intravenous injection of 4 and 100 nm-sized PEG-coated gold nanoparticles,” *Toxicol*. *Lett*., vol. 191, no. 1, pp. 96–102, 2009, doi: 10.1016/j.toxlet.2009.08.010 19695318

[58] Mobley W. H., “Intermediate linkages in the relationship between job satisfaction and employee turnover.,” *J*. *Appl*. *Psychol*., vol. 62, no. 2, pp. 237–240, 1977, doi: 10.1037/0021-9010.62.2.237

[59] Hulin C. L. and Hanisch K. A., “General Attitudes and Organizational Withdrawal: An Evalfile:///Users/karolinegranheim/Desktop/teori og notater/Prestasjonskultur i kunnskapsadhokratier.pdfuation of a Causal Modelfile:///Users/karolinegranheim/Desktop/teori og notater/Prestasjonskultur,” *J*. *Vocat*. *Behav*., vol. 39, pp. 110–128, 1991.

[60] Umamaheswari S. and Joyce S., “Antecedents and consequence of organizational commitment among employees of ceramic industries in India,” *J*. *Crit*. *Rev*., vol. 7, no. 6, pp. 20–22, 2020, doi: 10.31838/jcr.07.06.05

[61] De Gieter S., Hofmans J., and Pepermans R., “Revisiting the impact of job satisfaction and organizational commitment on nurse turnover intention: An individual differences analysis,” *Int*. *J*. *Nurs*. *Stud*., vol. 48, no. 12, pp. 1562–1569, 2011, doi: 10.1016/j.ijnurstu.2011.06.007 21821254

[62] Meyer J. P. and Herscovitch L., “Commitment in the workplace: Toward a general model,” *Hum*. *Resour*. *Manag*. *Rev*., vol. 11, no. 3, pp. 299–326, 2001, doi: 10.1016/S1053-4822(00)00053-X

[63] Ridwan M., Mulyani S. R., and Ali H., “Improving employee performance through perceived organizational support, organizational commitment and organizational citizenship behavior,” *Syst*. *Rev*. *Pharm*., vol. 11, no. 12, pp. 839–849, 2020, doi: 10.31838/srp.2020.5.123

[64] Tansky J. W. and Cohen D. J., “The relationship between organizational support, employee development, and organizational commitment: An empirical study,” *Hum*. *Resour*. *Dev*. *Q*., vol. 12, no. 3, pp. 285–300, 2001, doi: 10.1002/hrdq.15

[65] Jung K. B., Kang S. W., and Choi S. B., “Empowering leadership, risk-taking behavior, and employees’ commitment to organizational change: The mediated moderating role of task complexity,” *Sustain*., vol. 12, no. 6, 2020, doi: 10.3390/su12062340

[66] Kao T. and Kantor R., “Part Two: Total Rewards: From Clarity to Action,” *J*. *Total Reward*., vol. 13, no. 4, p. 32, 2004.

[67] A. Alrowwad, D. A. Almajali, R. Masa’Deh, B. Obeidat, and N. Aqqad, “The role of organizational commitment in enhancing organizational effectiveness,” *Proc. 33rd Int. Bus. Inf. Manag. Assoc. Conf. IBIMA 2019 Educ. Excell. Innov. Manag. through Vis. 2020*, no. April, pp. 9133–9154, 2009.

[68] Shin Y., Hur W. M., Park K., and Hwang H., “How managers’ job crafting reduces turnover intention: The mediating roles of role ambiguity and emotional exhaustion,” *Int*. *J*. *Environ*. *Res*. *Public Health*, vol. 17, no. 11, pp. 1–18, 2020, doi: 10.3390/ijerph17113972 32503324 PMC7312916

[69] Munir A. et al., “The impact of career planning and career satisfaction on employee’s turnover intention,” *Entrep*. *Sustain*. *Issues*, vol. 8, no. 1, pp. 218–232, 2020.

[70] Zahari A. E., Supriyati Y., and Santoso B., “The Influence of Compensation and Career Development Mediated Through Employee Engagement Toward Turnover Intention of The Permanent Officers Employees at The Head Office of PT Bank Syariah Mandiri,” *J*. *Int*. *Conf*. *Proc*., vol. 3, no. 1, pp. 22–40, 2020, doi: 10.32535/jicp.v2i4.777

[71] Batt R., “Managing customer services: Human resource practices, quit rates, and sales growth,” *Acad*. *Manag*. *J*., vol. 45, no. 3, pp. 587–597, 2002, doi: 10.2307/3069383

[72] Shahzad K., Hussain S., Bashir S., Chishti A. F., and Nasir Z. M., Organizational Environment, Job Satisfaction and Career Growth Opportunities: A Link to Employee Turnover Intentions in Public Sector of Pakistan, vol. 2, no. 9. 2011.

[73] Lee J., Kim S. B., Chae C., and Lee J., “Career Growth Opportunity on Turnover Intention: The Mediating Role of Organizational Commitment in Multinational Corporations,” *Int*. *J*. *Hum*. *Resour*. *Stud*., vol. 9, no. 4, p. 1, 2019, doi: 10.5296/ijhrs.v9i4.15245

[74] Mengstie M. M., “Perceived organizational justice and turnover intention among hospital healthcare workers,” *BMC Psychol*., vol. 8, no. 1, p. 19, 2020, doi: 10.1186/s40359-020-0387-8 32087743 PMC7036232

[75] Kuvaas B., “Employee ownership and affective organizational commitment: Employees’ perceptions of fairness and their preference for company shares over cash,” *Scand*. *J*. *Manag*., vol. 19, no. 2, pp. 193–212, 2003, doi: 10.1016/S0956-5221(01)00044-6

[76] Van Wyk A. E., Swarts I., and Mukonza C., “The Influence of the Implementation of Job Rotation on Employees’ Perceived Job Satisfaction,” *Int*. *J*. *Bus*. *Manag*., vol. 13, no. 11, p. 89, 2018, doi: 10.5539/ijbm.v13n11p89

[77] Igbaria M. and Wormley W. M., “Organizational experiences and career success of MIS professionals and managers: An examination of race differences,” MIS Q., pp. 507–529, 1992.

[78] IRABOR I. E. and OKOLIE U. C., “A Review of Employees’ Job Satisfaction and its Affect on their Retention,” *Ann*. *Spiru Haret Univ*. *Econ*. *Ser*., vol. 19, no. 2, pp. 93–114, 2019, doi: 10.26458/1924

[79] Hee O. C., Ying Y. H., Kowang T. O., Rizal A. M., and Ping L. L., “Succession management practices and employee retention in the property industry: Evidence from Malaysia,” *Int*. *J*. *Sci*. *Technol*. *Res*., vol. 8, no. 10, pp. 1409–1412, 2019.

[80] S. S. & Y. Li, “Human resource management practices on exit, voice, loyalty, and neglect: Organizational commitment as a mediator,” Int. J. Hum. Resour. *Manag*., vol. 23, no. 8, pp. 1705–1716, 2012, doi: 10.1080/09585192.2011.580099

[81] Weng Q. and McElroy J. C., “Organizational career growth, affective occupational commitment and turnover intentions,” *J*. *Vocat*. *Behav*., vol. 80, no. 2, pp. 256–265, 2012, doi: 10.1016/j.jvb.2012.01.014

[82] & Simiyu M. M., B. K., “Influence of career development on employee commitment: A case study of Masinde Muliro University of Science and Technology,” *Strateg*. *J*. *Bus*. *Chang*. *Manag*., vol. 6, no. 1, pp. 555–572, 2019

[83] Napitupulu S., Haryono T., Laksmi Riani A., Sawitri H. S. R., and Harsono M., “The impact of career development on employee performance: an empirical study of the public sector in Indonesia,” *Int*. *Rev*. *Public Adm*., vol. 22, no. 3, pp. 276–299, 2017, doi: 10.1080/12294659.2017.1368003

[84] F. P. & R. M. K. H. Nurita Juhdi, “HR practices and turnover intention: the mediating roles of organizational commitment and organizational engagement in a selected region in Malaysia,” Int. J. Hum. *Resour*. *Manag*., vol. 24, no. 15, pp. 3002–3019, 2013.

[85] Wu P. C. and Chaturvedi S., “The role of procedural justice and power distance in the relationship between high performance work systems and employee attitudes: A multilevel perspective,” *J*. *Manage*., vol. 35, no. 5, pp. 1228–1247, 2009, doi: 10.1177/0149206308331097

[86] Ballout H. I., “Career commitment and career success: Moderating role of self-efficacy,” *Career Dev*. *Int*., vol. 14, no. 7, pp. 655–670, 2009, doi: 10.1108/13620430911005708

[87] Đorđević B., Ivanović-Đukić M., Lepojević V., and Milanović S., “The impact of employees’ commitment on organizational performances,” *Strateg*. *Manag*., vol. 25, no. 3, pp. 28–37, 2020, doi: 10.5937/straman2003028d

[88] Loan L. T. M., “The influence of organizational commitment on employees’ job performance: The mediating role of job satisfaction,” *Manag*. *Sci*. *Lett*., vol. 10, no. 14, pp. 3307–3312, 2020, doi: 10.5267/j.msl.2020.6.007

[89] Jiang K., Lepak D. P., Hu J., and Baer J. C., “How does human resource management influence organizational outcomes? A meta-analytic investigation of mediating mechanisms,” *Acad*. *Manag*. *J*., vol. 55, no. 6, pp. 1264–1294, 2012, doi: 10.5465/amj.2011.0088

[90] Haryono S., Supardi S., and Udin U., “The effect of training and job promotion on work motivation and its implications on job performance: Evidence from Indonesia,” *Manag*. *Sci*. *Lett*., vol. 10, no. 9, pp. 2107–2112, 2020, doi: 10.5267/j.msl.2020.1.019

[91] Abu Hassan Asaari M. H., Mat Desa N., and Subramaniam L., “Influence of Salary, Promotion, and Recognition toward Work Motivation among Government Trade Agency Employees,” *Int*. *J*. *Bus*. *Manag*., vol. 14, no. 4, p. 48, 2019, doi: 10.5539/ijbm.v14n4p48

[92] Danish R. Q. and Usman A., “Impact of Reward and Recognition on Job Satisfaction and Motivation: An Empirical study from Pakistan,” *Int*. *J*. *Bus*. *Manag*., vol. 5, no. 2, pp. 159–167, 2010, doi: 10.5539/ijbm.v5n2p159

[93] Den Hartog D. N. and Verburg R. M., “High performance work systems, organisational culture and firm effectiveness,” *Hum*. *Resour*. *Manag*. *J*., vol. 14, no. 1, pp. 55–78, 2004, doi: 10.1111/j.1748-8583.2004.tb00112.x

[94] Hussain A., Khan M. A., and Hussain J., “Interplay of Organizational Commitment and Turnover Intention in Academic Sector,” *Rev*. *Econ*. *Dev*. *Stud*., vol. 6, no. 2, pp. 501–512, 2020, doi: 10.47067/reads.v6i2.218

[95] Zhou H., Long L. R., and Wang Y. Q., “What is the most important predictor of employees’turnover intention in chinese call centre: Job satisfaction, organisational commitment or career commitment?,” *Int*. *J*. *Serv*. *Technol*. *Manag*., vol. 12, no. 2, pp. 129–145, 2009, doi: 10.1504/IJSTM.2009.025231

[96] Cooper-Hakim A. and Viswesvaran C., “The construct of work commitment: Testing an integrative framework,” *Psychol*. *Bull*., vol. 131, no. 2, pp. 241–259, 2005, doi: 10.1037/0033-2909.131.2.241 15740421

[97] Ninroon P., Khlungsaeng W., Boonying J., and Vaiyavuth R., “Identifying the predictors of the turnover intentions: A case of Thailand pharmaceutical companies,” *Syst*. *Rev*. *Pharm*., vol. 11, no. 3, pp. 134–143, 2020, doi: 10.5530/srp.2020.3.15

[98] Choong Y.-O., Wong K.-L., and Lau T.-C., “Organizational Commitment: An Empirical Investigation on the Academician of Malaysian Private Universities,” *Bus*. *Econ*. *Res*. *J*., vol. 3, no. 2, pp. 51–64, 2012.

[99] Dick G. and Metcalfe B., “Managerial factors and organisational commitment: A comparative study of police officers and civilian staff,” *Int*. *J*. *Public Sect*. *Manag*., vol. 14, no. 2–3, pp. 111–128, 2001, doi: 10.1108/09513550110387336

[100] Hom P. W. et al., “Explaining employment relationships with social exchange and job embeddedness.,” *J*. *Appl*. *Psychol*., vol. 94, no. 2, p. 277, 2009. doi: 10.1037/a0013453 19271791

[101] March J. G., “The study of organizations and organizing since 1945,” *Organ*. *Stud*., vol. 28, no. 1, pp. 9–19, 2007, doi: 10.1177/0170840607075277

[102] Meyer J. P., Allen N. J., and Smith C. A., “Commitment to Organizations and Occupations: Extension and Test of a Three-Component Conceptualization,” *J*. *Appl*. *Psychol*., vol. 78, no. 4, pp. 538–551, 1993, doi: 10.1037/0021-9010.78.4.538

[103] Meyer J. P. and Parfyonova N. M., “Normative commitment in the workplace: A theoretical analysis and re-conceptualization,” *Hum*. *Resour*. *Manag*. *Rev*., vol. 20, no. 4, pp. 283–294, 2010, doi: 10.1016/j.hrmr.2009.09.001

[104] San Martín H. and Rodríguez del Bosque I. A., “Exploring the cognitive-affective nature of destination image and the role of psychological factors in its formation,” *Tour*. *Manag*., vol. 29, no. 2, pp. 263–277, 2008, doi: 10.1016/j.tourman.2007.03.012

[105] Yucel I., McMillan A., and Richard O. C., “Does CEO transformational leadership influence top executive normative commitment?,” *J*. *Bus*. *Res*., vol. 67, no. 6, pp. 1170–1177, 2014, doi: 10.1016/j.jbusres.2013.05.005

[106] Ahmad K. Z. and Bakar R. A., “The association between training and organizational commitment among white-collar workers in Malaysia,” *Int*. *J*. *Train*. *Dev*., vol. 7, no. 3, pp. 166–185, 2003, doi: 10.1111/1468-2419.00179

[107] Al-shahwan D., “Research and Development GAP at Countries of Middle East and North Africa and Its Arab Reflections: Turkey an Example,” *TANMIYAT AL-RAFIDAIN*, vol. 28, no. 83, pp. 175–203, 2006.

[108] Al Wahshi A. S., “Human resource planning practices in the Omani Public Sector: An exploratory study in the Ministry of Education in the Sultanate ducation in the Sultanate of Oman,” 2016.

[109] Lepak L. H. N. D. P., “EMPLOYEE ATTRIBUTIONS OF THE ‘WHY’ OF HR PRACTICES: THEIR EFFECTS ON EMPLOYEE ATTITUDES AND BEHAVIORS, AND CUSTOMER SATISFACTION,” *Pers*. *Psychol*., vol. 61, pp. 503–545, 2008.

[110] Rajasekar J. and Khan S., “Training and Development Function in Omani Public Sector Organizations: A Critical Evaluation,” *J*. *Appl*. *Bus*. *Econ*., vol. 14, no. 2, pp. 37–52, 2013.

[111] Bawaba A., “GCC Countries building for the future with efforts on recruitment and training,” *Al Bawaba*, p. 6, 2009.

[112] Adler N. J. and Gundersen A., *International dimensions of organizational behavior*. Mason, Ohio: Thomson/South-Western, 2008.

[113] Fowler F. J. Jr, *Survey research methods*. Sage publications, 2013.

[114] Crowther D. and Lancaster G., *Research methods*. Routledge, 2012.

[115] Sekaran U. and Bougie R., *Research methods for business*: *A skill building approach*. John Wiley & Sons, 2016.

[116] Hair J. F., Black W. C., Babin B. J., and Anderson R. E., “Multivariate data analysis: Pearson new international edition,” *Essex Pearson Educ*. *Ltd*., vol. 1, p. 2, 2014.

[117] Saunders M., Lewis P., and Thornhill A., *Chapter 4*: *Understanding research philosophy and approaches to theory development*, no. January. 2019.

[118] Green S B, “How Many Subjects Does It Take To Do A Regression Analysis,” *Multivariate Behav*. *Res*., vol. 26, no. 3, pp. 499–510, 1991, doi: 10.1207/s15327906mbr2603_7 26776715

[119] Robert V D. W. M.Krejcie., “Activities of human RRP6 and structure of the human RRP6 catalytic domain,” *Educ*. *Psychol*. *Meas*., vol. 30, pp. 607–610, 1970, doi: 10.1261/rna.2763111 21705430 PMC3153979

[120] Hair J. F. Jr, Page M., and Brunsveld N., *Essentials of business research methods*. Routledge, 2019.

[121] and C P. H. M. C. (eds. Seashore Stanley E., Lawler Edward E. III, Assessing Organizational Change. A Guide to Methods, Measures, and Practices. New York: Wiley, 1983.

[122] Richard T R. M. S. Mowday., “Experimental methanol toxicity in the primate: Analysis of metabolic acidosis,” J. vactional Behav., vol. 14, pp. 224–247, 1979, doi: 10.1016/0041-008X(75)90174-X

[123] Mcdonald K. S. and Hite L. M., “Reviving the Relevance of Career Development in Human Resource Development,” *Hum*. *Resour*. *Dev*. *Rev*., vol. 4, no. 4, pp. 418–439, 2005, doi: 10.1177/1534484305281006

[124] Huselid M. A. and Day N. E., “Organizational Commitment, Job Involvement, and Turnover: A Substantive and Methodological Analysis,” *J*. *Appl*. *Psychol*., vol. 76, no. 3, pp. 380–391, 1991, doi: 10.1037/0021-9010.76.3.380

[125] Khan I., Khan F., Khan H., Nawaz A., and Bakht Yar N., “Determining the Demographic impacts on the Organizational Commitment of Academicians in the HEIs of DCs like Pakistan,” *Eur*. *J*. *Sustain*. *Dev*., vol. 2, no. 2, pp. 117–130, 2013, doi: 10.14207/ejsd.2013.v2n2p117

[126] Chang E., “Career commitment as a complex moderator of organizational commitment and turnover intention,” *Hum*. *Relations*, vol. 52, no. 10, pp. 1257–1278, 1999, doi: 10.1177/001872679905201002

[127] Lum L., Kervin J., Clark K., Reid F., and Sirola W., “Explaining nursing turnover intent: Job satisfaction, pay satisfaction, or organizational commitment?,” *J*. *Organ*. *Behav*., vol. 19, no. 3, pp. 305–320, 1998, doi: 10.1002/(SICI)1099-1379(199805)19:3&lt;305::AID-JOB843&gt;3.0.CO;2-N

[128] Mathieu J. E. and Zajac D. M., “A review and meta-analysis of the antecedents, correlates, and consequences of organizational commitment.,” *Psychol*. *Bull*., vol. 108, no. 2, pp. 171–194, 1990, doi: 10.1037/0033-2909.108.2.171

[129] Meyer J. P., Stanley D. J., Herscovitch L., and Topolnytsky L., “Affective, continuance, and normative commitment to the organization: A meta-analysis of antecedents, correlates, and consequences,” *J*. *Vocat*. *Behav*., vol. 61, no. 1, pp. 20–52, 2002, doi: 10.1006/jvbe.2001.1842

[130] Millard S., “North Australian Pastoral Company (NAPCO) Experiences with composites,” *Armidale Feed. steer Sch*., no. S3, pp. 131–133, 2003.

[131] Guchait P. and Cho S., “The impact of human resource management practices on intention to leave of employees in the service industry in India: The mediating role of organizational commitment,” *Int*. *J*. *Hum*. *Resour*. *Manag*., vol. 21, no. 8, pp. 1228–1247, 2010, doi: 10.1080/09585192.2010.483845

[132] Porter L. W. and Steers R. M., “Organizational, work, and personal factors in employee turnover and absenteeism.,” *Psychol*. *Bull*., vol. 80, no. 2, pp. 151–176, 1973, doi: 10.1037/h0034829

[133] Byrne B. M., *Structural equation modeling with AMOS: basic concepts, applications, and programming (multivariate applications series)*, vol. 396. 2010.

[134] Awang Z., *Introduction to Structural Dynamics*, 4th Editio. Kelantan: University Technology MARA Press, 2012.

[135] Kline R. B., *Principles and Practices of Structureal Equation Modeling*, THIRD EDIT. New York London: The Guilford Press, 2011.

[136] Santos J. R. A., “Cronbach’s alpha: A tool for assessing the reliability of scales,” *J*. *Ext*., vol. 37, no. 2, pp. 88–92, 1999.

[137] Cronbach L. J., “Coefficient alpha and the internal structure of tests,” *Psychometrika*, vol. 16, no. 3, pp. 297–334, 1951, doi: 10.1007/BF02310555

[138] Raykov T., “Behavioral scale reliability and measurement invariance evaluation using latent variable modeling,” *Behav*. *Ther*., vol. 35, no. 2, pp. 299–331, 2004, doi: 10.1016/S0005-7894(04)80041-8

[139] Bollen K. A. and Jackman R. W., “Regression diagnostics: An expository treatment of outliers and influential cases,” *Sociol*. *Methods Res*., vol. 13, no. 4, pp. 510–542, 1985, doi: 10.1177/0049124185013004004

[140] De Arcangelis G., “Structural Equations with Latent Variables.” JSTOR, 1993.

[141] Byrne B. M., *Structural equation modeling with AMOS: Basic concepts, applications, and programming* 2nd Edition, 2nd Editio. CRC Press, 2009.

[142] Barrett P., “Structural equation modelling: Adjudging model fit,” *Pers*. *Individ*. *Dif*., vol. 42, no. 5, pp. 815–824, 2007, doi: 10.1016/j.paid.2006.09.018

[143] Bartlett K. R., “The relationship between training and organizational commitment: A study in the health care field,” *Hum*. *Resour*. *Dev*. *Q*., vol. 12, no. 4, pp. 335–352, 2001, doi: 10.1002/hrdq.1001

[144] news Agency O., “Gulf News Hails Omani Women’s Role in Public, Private Sectors,” *Agency, Oman news*, p. 6, 2020.

[145] Bentler P.M., “Multivariate analysis,” *Annu*. *Rev*. *Psychol*., vol. 31, pp. 419–456, 1980, doi: 10.1007/978-3-319-77203-5_8

[146] Davey A., *Statistical power analysis with missing data*: *A structural equation modeling approach*. Routledge, 2009.

[147] Sonia S. M., “Relational and economic antecedents of organisational commitment,” *Pers*. *Rev*., vol. 37, no. 6, pp. 589–608, Jan. 2008, doi: 10.1108/00483480810906856

[148] Blinman P. L. et al., “The shortage of medical oncologists: The Australian medical oncologist workforce study,” *Med*. *J*. *Aust*., vol. 196, no. 1, pp. 58–61, 2012, doi: 10.5694/mja11.10363 22256937

[149] Natalie J J. P. M. Allen., “The measurement and antecedents of affective, continuance and normative commitment to the organization,” *J*. *Occup*. *Psychol*., vol. 63, pp. 1–18, 1990.

[150] Newman A., Thanacoody R., and Hui W., “The impact of employee perceptions of training on organizational commitment and turnover intentions: A study of multinationals in the Chinese service sector,” *Int*. *J*. *Hum*. *Resour*. *Manag*., vol. 22, no. 8, pp. 1765–1787, 2011, doi: 10.1080/09585192.2011.565667

[151] Vandenberghe C., Panaccio A., and Ben Ayed A. K., “Continuance commitment and turnover: Examining the moderating role of negative affectivity and risk aversion,” *J*. *Occup*. *Organ*. *Psychol*., vol. 84, no. 2, pp. 403–424, 2011, doi: 10.1348/096317910X491848

