# Supplementary material for: Role of organizational commitment in career growth and turnover intention in public sector of Oman
Source: PLoS One. 2022 May 12;17(5):e0265535. doi: 10.1371/journal.pone.0265535 (PMC9098061; doi:10.1371/journal.pone.0265535)
Supplement: S1 Appendix — (DOCX) [file pone.0265535.s001.docx]

Appendix 1

**Mahalanobis Distance (Observations Farthest from the Centroid)**

| **Observation number** | **Mahalanobis d-squared** | **p1** | **p2** |
| --- | --- | --- | --- |
| 320 | 108.498 | .000 | .000 |
| 326 | 90.976 | .000 | .000 |
| 293 | 90.923 | .000 | .000 |
| 310 | 84.506 | .000 | .000 |
| 311 | 81.443 | .000 | .000 |
| 312 | 80.577 | .000 | .000 |
| 282 | 78.069 | .000 | .000 |
| 290 | 76.898 | .000 | .000 |
| 313 | 75.929 | .000 | .000 |
| 274 | 74.366 | .000 | .000 |
| 323 | 72.057 | .000 | .000 |
| 271 | 70.338 | .000 | .000 |
| 285 | 69.220 | .000 | .000 |
| 308 | 67.422 | .000 | .000 |
| 325 | 63.824 | .000 | .000 |
| 316 | 63.430 | .001 | .000 |
| 283 | 61.440 | .001 | .000 |
| 269 | 60.670 | .001 | .000 |
| 162 | 60.475 | .001 | .000 |
| 309 | 60.407 | .001 | .000 |
| 307 | 60.059 | .001 | .000 |
| 322 | 58.298 | .002 | .000 |
| 284 | 56.726 | .003 | .000 |
| 292 | 56.480 | .003 | .000 |
| 299 | 55.925 | .004 | .000 |
| 288 | 54.467 | .006 | .000 |
| 246 | 53.344 | .008 | .000 |
| 302 | 51.871 | .011 | .000 |
| 83 | 51.769 | .011 | .000 |
| 306 | 51.699 | .011 | .000 |
| 240 | 51.598 | .012 | .000 |
| 272 | 51.356 | .012 | .000 |
| 314 | 51.134 | .013 | .000 |
| 239 | 49.991 | .017 | .000 |
| 281 | 49.545 | .019 | .000 |
| 321 | 48.555 | .023 | .000 |
| 176 | 48.167 | .025 | .000 |
| 11 | 47.986 | .026 | .000 |
| 202 | 47.555 | .029 | .000 |
| 8 | 46.831 | .034 | .000 |
| 43 | 46.712 | .035 | .000 |
| 101 | 46.129 | .039 | .000 |
| 63 | 45.981 | .041 | .000 |
| 241 | 45.743 | .043 | .000 |
| 7 | 45.108 | .049 | .000 |
| 262 | 44.965 | .050 | .000 |
| 276 | 44.744 | .053 | .000 |
| 295 | 44.397 | .056 | .000 |
| 234 | 43.818 | .063 | .000 |
| 68 | 43.548 | .067 | .000 |
| 2 | 43.523 | .067 | .000 |
| 143 | 43.232 | .071 | .000 |
| 278 | 42.894 | .076 | .000 |
| 250 | 42.402 | .083 | .000 |
| 296 | 42.169 | .087 | .000 |
| 318 | 41.763 | .094 | .000 |
| 14 | 41.436 | .100 | .000 |
| 298 | 41.409 | .100 | .000 |
| 15 | 41.371 | .101 | .000 |
| 280 | 41.347 | .101 | .000 |
| 291 | 41.221 | .104 | .000 |
| 13 | 41.127 | .106 | .000 |
| 9 | 40.540 | .117 | .000 |
| 198 | 40.336 | .122 | .000 |
| 255 | 40.212 | .124 | .000 |
| 315 | 39.949 | .130 | .000 |
| 329 | 39.875 | .132 | .000 |
| 4 | 39.819 | .133 | .000 |
| 327 | 39.791 | .134 | .000 |
| 223 | 39.699 | .136 | .000 |
| 300 | 39.431 | .142 | .000 |
| 222 | 39.366 | .144 | .000 |
| 275 | 39.348 | .144 | .000 |
| 257 | 39.208 | .148 | .000 |
| 49 | 38.989 | .153 | .000 |
| 204 | 38.732 | .160 | .001 |
| 30 | 38.659 | .162 | .000 |
| 211 | 38.531 | .166 | .001 |
| 16 | 38.336 | .171 | .001 |
| 277 | 38.317 | .172 | .001 |
| 242 | 38.200 | .175 | .001 |
| 199 | 38.018 | .180 | .001 |
| 10 | 37.819 | .186 | .002 |
| 205 | 37.789 | .187 | .001 |
| 66 | 37.667 | .191 | .002 |
| 54 | 37.538 | .194 | .002 |
| 254 | 37.222 | .204 | .005 |
| 258 | 37.069 | .209 | .007 |
| 213 | 36.513 | .228 | .039 |
| 268 | 36.216 | .238 | .076 |
| 180 | 36.122 | .241 | .079 |
| 212 | 36.106 | .242 | .065 |
| 67 | 36.049 | .244 | .061 |
| 245 | 35.901 | .249 | .074 |
| 218 | 35.892 | .250 | .060 |
| 73 | 35.854 | .251 | .053 |
| 18 | 35.821 | .252 | .046 |
| 42 | 35.768 | .254 | .042 |
| 270 | 35.694 | .257 | .041 |
| 120 | 35.553 | .262 | .051 |
